# Highly efficient heterogeneous photo-Fenton BiOCl/MIL-100(Fe) nanoscaled hybrid catalysts prepared by green one-step coprecipitation for degradation of organic contaminants†

**DOI:** 10.1039/d1ra06549a

**Published:** 2021-10-01

**Authors:** Doufeng Wu, Jiantang Jiang, Nini Tian, Mei Wang, Jing Huang, Deyou Yu, Minghua Wu, Huagang Ni, Peng Ye

**Affiliations:** Key Laboratory of Advanced Textile Materials and Manufacturing Technology of Education Ministry, Zhejiang Sci-Tech University Hangzhou 310018 P. R. China nhuag@163.com dfwu@zstu.edu.cn; Engineering Research Center for Eco-Dyeing and Finishing of Textiles, Key Laboratory of Advanced Textile Materials & Manufacturing Technology, Ministry of Education, Zhejiang Sci-Tech University Hangzhou 310018 P. R. China jiantangjiang@163.com; Key Laboratory of Surface & Interface Science of Polymer Materials of Zhejiang Province, Department of Chemistry, Zhejiang Sci-Tech University Hangzhou 310018 P. R. China

## Abstract

An excellent heterojunction structure is vital for the improvement of photocatalytic performance. In this study, BiOCl/MIL-100(Fe) hybrid composites were prepared *via* a one-pot coprecipitation method for the first time. The prepared materials were characterized and then used as a photo-Fenton catalyst for the removal of organic pollutants in wastewater. The BiOCl/MIL-100(Fe) hybrid exhibited better photo-Fenton activity than MIL-100(Fe) and BiOCl for RhB degradation; in particular, the hybrid with 50% Bi molar concentration showed the highest efficiency. The excellent performance can be ascribed to the presence of coordinatively unsaturated iron centers, abundant Lewis acid sites, fast H_2_O_2_ activation, and efficient carrier separation on BiOCl nanosheets due to the high charge carrier mobility of the nanosheets. The photo-Fenton mechanism was studied, and the results indicated that ˙OH and h^+^ were the main active species for organic pollutant degradation. The coprecipitation-based hybridization approach presented in this paper opens up an avenue for the sustainable fabrication of photo-Fenton catalysts with abundant coordinatively unsaturated metal centers and efficient electron–hole separation capacity.

## Abstract 2

An excellent heterojunction structure is vital for the improvement of photocatalytic performance.

## Graphical abstract

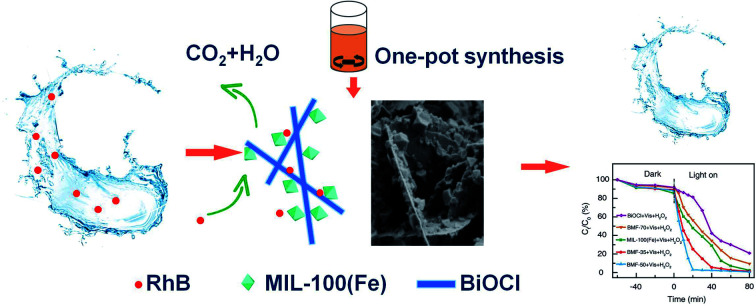

## 1 Introduction

In recent years, environmental pollution, especially water pollution, has been a critical problem worldwide. Industrial effluent discharged into water is considered the main cause of environmental pollution. These effluents generally contain massive organic contaminants, such as dyes, which are toxic and carcinogenic.^1^ The presence of dye wastewater in waterbodies directly threatens the health of humans and other biological organisms; hence, how to effectively remove these persistent organic pollutants in wastewater is a vital issue.^2^ Advanced oxidation processes, one of the effective and promising wastewater treatment technologies, have been used for removing various persistent organic pollutants from wastewater through the generation of reactive oxygen species.^3,4^ Among the advanced oxidation processes, the Fenton reaction (Fe^3+^/Fe^2+^ + H_2_O_2_) has drawn much attention because of its higher ˙OH generation rate, low cost, and simplicity. However, the traditional homogeneous Fenton reaction is associated with several inherent drawbacks, such as Fe-containing sludge production and loss of catalyst. Hence, heterogeneous Fenton-like reaction using insoluble catalysts has been developed to overcome the above problems.^5^ Various iron oxides and Fe-immobilized materials have been used for wastewater treatment;^6,7^ however, these catalysts show low activity and severe iron leaching. Accordingly, developing new types of heterogeneous catalysts with high catalytic activity and durability has been a primary pursuit.^8^ For the traditional heterogeneous Fenton reaction, the redox cycling of Fe(iii)/Fe(ii) and Fe^3+^/Fe^2+^ by H_2_O_2_ is critical to keep the Fenton reactions continuous. Meanwhile, the reduction of Fe^3+^/Fe(iii) by H_2_O_2_ is always the rate-limiting step determining the overall efficiency of the whole Fenton reactions.^9^ Therefore, how to accelerate the redox cycling of Fe(iii)/Fe(ii) and promote the H_2_O_2_ utilization efficiency in traditional heterogeneous Fenton reactions is a core issue that has motivated researchers to design more effective heterogeneous Fenton catalysts and reaction strategies.

Iron-based metal–organic frameworks (MOFs) constructed from metal ions or clusters and organic ligands have been widely used in wastewater treatment as heterogeneous Fenton-like catalysts because of their specific textural properties, including large surface areas, porous structures, and wide distribution of single iron sites; these properties endow the Fe-based MOFs with abundant exposed active sites and are favorable to reactant transfer. Among the MOFs, MILs(Fe), such as MIL-88A(Fe), MIL-88B(Fe), MIL-53(Fe), MIL-100(Fe), and MIL-101(Fe), are constructed from carboxylate ligands (fumaric acid, benzene-1,4-dicarboxylic acid (H_2_BDC), benzene-1,3,5-tricarboxylic acid (H_3_BTC)) and iron(iii).^10^ However, the pure Fe-MOFs show weak performances in activating H_2_O_2_ to degrade dye, and the reaction mechanism is unclear.^11^ Metal nodes in the majority of MOFs are completely occupied by coordinated organic linkers, which reduce the number of Lewis acid sites and make them unavailable for H_2_O_2_ activation.^11^ The coordinatively unsaturated metal centers (CUCs), that is, Lewis acid sites, are the real active centers. Hence, the incorporation of coordinatively unsaturated metal sites (CUSs) is a feasible strategy to realize more exposed active metal sites for efficient Fenton activity.^12,13^ In addition, Fenton-like systems under visible light processes have been found to accelerate the reduction of Fe^3+^ to Fe^2+^ and exhibit high-energy efficiency and relatively high mineralization.^14,15^ Dual-functional catalysts possessing photo-Fenton activity are the key to this chemical process. Each metal–oxo cluster in MOFs can be identified as a single quantum dot that can serve as a light absorber, charge generator, and catalytic site, behaving like a small semiconductor.^16,17^ In one study, owing to the unique characteristics of visible light absorption, electron transfer from O(ii) to Fe(iii) occurred under visible-light irradiation, leading to the acceleration of the Fe(ii)/Fe(iii) cycle.^18^ However, the limited light absorption and poor charge separation properties of Fe-based MOFs have been found to significantly reduce their performance in photo-Fenton coupling reactions and result in the incomplete mineralization of organic pollutant molecules.^19^

Hence, various strategies have been adopted to improve the catalytic performance of Fe-MOFs in photo-Fenton processes for organic pollutant removal. For example, in a previous study, ligand defect-containing NH_2_-MIL-88B(Fe) exhibited enhanced photo-Fenton catalytic performance. The modified MIL-88(Fe) exhibited 7.3 times higher adsorption rate and 5.5 times higher catalytic rate for acetamiprid than the pristine MIL-88(Fe) did. Moreover, the presence of the ligand defects on NH_2_-MIL-88B(Fe) could introduce a large number of monodentate ligands and ligand vacancies in MOFs to promote light absorption and electron–hole separation capacity for photocatalysis. Also, the presence of the ligand defects could increase the number of Fe Lewis acid sites to improve the redox capacity of Fe^2+^/Fe^3+^ for Fenton catalysis.^20^ In one study, researchers regulated the electronegativity of Fe–O clusters in MIL-53(Fe) to guide the transfer of photogenerated electrons. The modified MIL-53(Fe) achieved improved thiamethoxam removal rate. The modified MIL-53(Fe) had a lower Fe^II^/Fe^III^ ratio, which reduced the electron density around Fe atoms. The Fe^III^ with higher electronegativity has a greater ability to attract negative electrons, which can reduce positron annihilation rate and increase positron lifetime.^21^ Bimetallic MOFs have also been found to exhibit highly efficient photo-Fenton degradation of organic pollutants under visible-light irradiation.^22,23^

Fabricating heterostructure materials by integrating MOFs with other functionalized materials is another strategy for improving the photocatalytic performance of MOFs. Compared with pure MOFs, MOF-based heterostructure materials have been found to show lower electron–hole recombination rates and higher visible-light adsorption regions.^24^ Heterostructure materials such as Fe-based MOFs/g-C_3_N_4_,^25^ MIL-88A(Fe)/GO composites,^26^ needle-shaped 1T-MoS_2_@MIL-53(Fe) composites,^27^ bismuth ferrite/MIL-53(Fe) nanocomposite,^28^ and CUCs-MIL-88B(Fe)/Ti_3_C_2_ (ref. 29) have been found to show enhanced photocatalytic activity because of the formation of a compact and uniform interfacial contact between the MOFs and other semiconductors; the interfacial contact accelerates the separation of the photoinduced charges and decreases the electron–hole recombination rate. Layered bismuth oxyhalides (BiOX, X = Cl, Br, I), which are promising photocatalysts,^30,31^ have attracted increasing attention owing to their indirect charge transition characteristic, more active-site exposure, and efficient separation of photoinduced electron–hole pairs. Moreover, MOFs and BiOX composites with improved catalytic degradation performance for organic pollutants have been developed. For example, in one study, BiOBr/NH_2_-MIL-125(Ti) composites and BiOBr/UiO-66 were prepared to degrade RhB and atrazine, respectively, under visible light.^32,33^ In another study, BiOBr/MIL-53(Fe) hybrid photocatalysts prepared *via* coprecipitation were used to decompose rhodamine B (RhB) and carbamazepine. All the hybrids exhibited better catalytic performance than the pristine BiOBr. The incorporated MIL-53(Fe) not only formed a heterojunction with BiOBr to inhibit the recombination of the photoinduced electron–hole pairs but also utilized the visible light more effectively.^1^ In another study, the semiconductor heterojunction of an MIL-53(Fe)/BiOCl composite prepared *via* a solvothermal reaction could accelerate the photoreactivity of BiOCl under visible-light irradiation and showed higher RhB degradation rate and total organic carbon (TOC) removal efficiency in the presence of persulfate.^34^ However, the use of single BiOCl as a photocatalyst will present poor photocatalytic performance under visible light because of its wide band gap and fast combination frequency of photoexcited carriers. According to previous research, fabricating heterostructure BiOCl/Fe-MOFs hybrids may remarkably improve the photo-Fenton catalytic activity to remove organic pollutants under visible light.

Furthermore, MIL-100(Fe) with the chemical composition Fe_3_O(H_2_O)_2_(X)·{C_6_H_3_(COO)_3_}_2_·*n*H_2_O (X = OH^−^ or F^−^) (*n* = 14.5) has excellent chemical stability and water stability and is relatively easy to synthesize.^35^ In one study, the efficiency of heterogeneous Fenton processes based on the decomposition of H_2_O_2_ over Fe clusters of MIL-100(Fe) to generate ˙OH was significantly enhanced under visible-light irradiation.^36^ Nevertheless, almost all the reported hybrid materials composed of BiOX and Fe-MOFs were prepared *via* hydrothermal or solvothermal methods, or BiOX or Fe-MOFs were first prepared and then the composites were obtained. How to obtain the BiOX/Fe-MOFs through a feasible and sustainable preparation method is still challenging.

In this work, a series of novel BiOCl/MIL-100(Fe) nanohybrid materials was prepared through a feasible one-step coprecipitation method for the first time. The physical and chemical properties of BiOCl/MIL-100(Fe) were characterized, and the photo-Fenton performance of BiOCl/MIL-100(Fe) was measured by testing it against organic dye degradation in the presence of H_2_O_2_ under visible-light irradiation. The BiOCl/MIL-100(Fe) showed higher photo-Fenton activity, which is likely associated with the higher number of Lewis acid sites of MIL-100(Fe) and efficient carrier separation on BiOCl lamellar nanosheets due to the fast mobility of charge carriers, especially holes. Moreover, the possible mechanism for the photoreaction process of BiOCl/MIL-100(Fe)/H_2_O_2_ is proposed.

## 2 Experimental section

### 2.1 Materials

Iron(ii) chloride tetrahydrate (FeCl_2_·4H_2_O), trimesic acid(1,3,5-benzenetricarboxylic acid), bismuth trichloride (BiCl_3_), methylene blue (MB), and RhB were supplied by Shanghai Macklin Biochemical Co., Ltd. Other reagents, such as sodium hydroxide (NaOH), hydrochloric acid (HCl, 37 wt%), and hydrogen peroxide (H_2_O_2_, 30 wt%), were used as received from commercial suppliers without any further purification.

### 2.2 Preparation of MIL-100(Fe)

Here, MIL-100(Fe) was synthesized following a previously reported sustainable method but with slight modification.^35,37^ First, 0.912 g NaOH was dissolved in 48.6 mL water; then 1.676 g H_3_BTC was introduced into the aqueous NaOH, and the mixture was stirred at 65 °C to obtain clear solution A. Subsequently, FeCl_2_·4H_2_O (1.44 g) solid was added into 48.6 mL water, and the mixture was stirred under totally enclosed conditions to obtain a green clear solution, denoted as solution B. Then, solution A was added dropwise into solution B under magnetic stirring. The green mixture gradually turned brown after 6 h of stirring at room temperature. After a certain time, the supernatant liquid was discarded. Then, water was added to the mixture, and the mixture was stirred for a certain time, after which it was allowed to stand for some time, and the supernatant liquid was discarded again. This process was conducted several times. Finally, the solid was recovered *via* centrifugation and thoroughly washed, first with hot (70 °C) and cold (room temperature) water and finally with ethanol. Then, the material was vacuum-dried overnight at 65 °C.

### 2.3 Preparation of BiOCl/MIL-100(Fe) hybrids

In this study, bismuth oxychloride (BiOCl) was prepared as follows: first, 0.912 g NaOH was dissolved in 48.6 mL water. Then, 1.676 g H_3_BTC was introduced into the aqueous NaOH, and the resulting mixture was stirred at 65 °C to obtain clear solution A. Solid BiCl_3_ (2.283 g) was added into 48.6 mL water, and the mixture was stirred under enclosed conditions to obtain solution B. Then, solution A was added dropwise into solution B under magnetic stirring. The white mixture gradually turned flocculent after 6 h of stirring at room temperature. After a certain time, the supernatant liquid was dumped. Subsequently, water was added to the mixture, and the mixture was stirred for a certain time, after which it was allowed to stand for some time; then, the supernatant liquid was dumped again. This process was conducted several times. Finally, the solid was recovered *via* centrifugation and thoroughly washed, first with hot water (70 °C) and cold water (room temperature) and finally with ethanol. Then, the material was vacuum-dried at 65 °C for 24 h.

Here, BiOCl/MIL-100(Fe) hybrids were prepared *via* a one-pot coprecipitation method. First, 0.912 g (22.8 mmol) NaOH was dissolved in 48.6 mL water; then, 1.676 g (7.980 mmol) H_3_BTC was introduced into the aqueous NaOH, and the mixture was stirred at 65 °C to obtain clear solution A. Afterward, FeCl_2_·4H_2_O (0.867 g, 4.36 mmol) solid was added into 48.6 mL water and stirred under totally enclosed conditions to obtain a clear green solution, and 1.437 g (4.56 mmol) BiCl_3_ was added to the mixture, which was then stirred for 30 min at room temperature to obtain solution B. Then, solution A was added dropwise into solution B under magnetic stirring. The resulting mixture gradually turned light brown and flocculent after 6 h of stirring at room temperature. After a certain time, the supernatant liquid was dumped. Then, water was added to the mixture, and the mixture was stirred for a certain time, after which it was allowed to stand. Then, the supernatant liquid was dumped again. This process was conducted several times. Finally, the solid was recovered *via* centrifugation and thoroughly washed, first with hot water (70 °C) and cold water (room temperature) and finally with ethanol. Afterward, the material was vacuum-dried at 65 °C for 24 h. The feed molar ratios of BiCl_3_ and FeCl_2_·4H_2_O were varied to prepare BiOCl/MIL-100(Fe) hybrids of different Bi molar concentrations: 35%, 50%, and 70%, denoted as BMF-35, BMF-50, and BMF-70, respectively.

### 2.4 Characterization

The crystal structures of BiOCl/MIL-100(Fe) hybrids were characterized *via* powder X-ray diffraction (XRD) technique on a Dandong Radius DX-2700 X-ray diffractometer (China). The morphologies and microstructures of samples were observed *via* field-emission scanning electron microscopy (FESEM, Hitachi SU8020) and transmission electron microscopy (HR-TEM, JEM-2100F). The X-ray photoelectron spectroscopy (XPS) spectra were obtained using an X-ray photoelectron spectrometer (K Alpha, USA). The Brunauer–Emmett–Teller surface areas and pore structures of catalysts were determined from nitrogen adsorption–desorption isotherms (ASAP 2020 volumetric adsorption analyzer). Fourier-transform infrared (FTIR) spectra were obtained using the Thermo Nicolet Avatar 370 FTIR spectrometer. Ultraviolet-visible (UV-vis) diffuse reflectance spectroscopy (DRS) was conducted using the Shimadzu UV-2600 UV-vis spectrometer. The photoluminescence (PL) spectra of catalysts were recorded using Hitachi F-7000 fluorescence spectrophotometer. Transient photocurrent response and electrochemical impedance spectra were obtained using a CHI-660E electrochemical workstation in Shanghai, China. Total organic carbon analysis was performed on a TOC analyzer (polynitrogen/C3100 polynitrogen analyzer). Electron spin resonance (ESR) spectra were obtained using a Bruker A300 spectrometer with 5,5-dimethyl-1-pyrroline-*N*-oxide (DMPO) as the spin trap at 30 °C. The Lewis acid sites in the catalysts were identified and quantified based on the chemisorbed-pyridine FTIR (Py-FTIR) spectra. These PyFTIR spectra were recorded using a Nicolet iS10 FTIR spectrometer (Thermo Scientific, USA).

### 2.5 Catalyst tests

In this research, RhB and MB were selected to evaluate the catalyst performance. The photo-Fenton performances of as-prepared BiOCl/MIL-100(Fe) and other samples were analyzed. The experiment was conducted in a 50 mL cylindrical glass reactor equipped with an LSH-500 W xenon lamp as the light source and a 420 nm cutoff filter. In the experiment, the catalyst (80 mg L^−1^) was added into 40 mL of RhB solution (40 mg L^−1^) under continuous magnetic stirring for 1 h to obtain adsorption–desorption equilibrium in the dark at 30 °C. The pH values of the solutions of the samples were not adjusted (pH = 6.8). Before illumination, H_2_O_2_ (7.4 mmol L^−1^) was added into the suspension to initiate the reaction. Then, 2 mL suspension was extracted at a planned interval and filtered using a 0.22 μm membrane filter. The solution was immediately quenched with methanol, and then, the UV-vis spectra of the residual RhB were measured (TU-1901, Beijing Purkinje General Instrument Co., Ltd.). The obtained powder was centrifuged and washed several times with deionized water and ethanol after the supernatant was removed and then dried for 24 h at 60 °C. The dried powder was reused for the next cycle experiment. Each experiment was run at least three times.

## 3 Results

### 3.1 Characterization

**Fig. 1:**
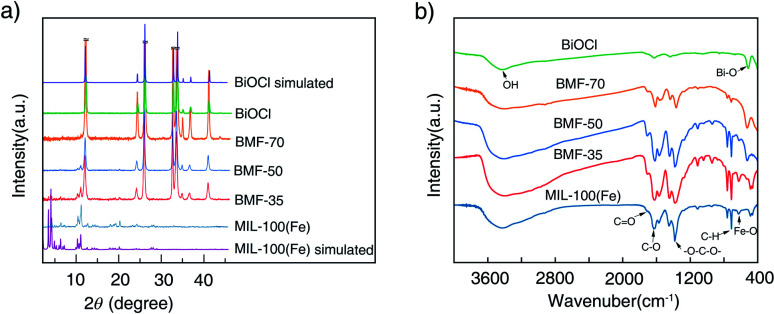
Powder X-ray diffraction patterns (a) and Fourier-transform infrared spectra (b) of BiOCl/MIL-100(Fe) hybrids.

The powder XRD patterns of the samples are shown in Fig. 1a. The diffraction peaks (2*θ*) of the synthesized MIL-100(Fe) are consistent with the peaks of simulated MIL-100(Fe) pattern.^38,39^ The spectra of the BiOCl can be indexed to the tetragonal crystallites of BiOCl (JCPDS 85-0861).^40^ In the spectra of the BiOCl/MIL-100(Fe) composites, that is, BMF-35 to BMF-70, with the increasing BiOCl content, the intensity of the diffraction peaks assigned to MIL-100(Fe) decreased. However, the (110) diffraction peak (2*θ* = 32.5°) of BiOCl in pure BiOCl was stronger than the (102) diffraction peak in the BiOCl/MIL-100(Fe) hybrid, suggesting that BiOCl preferably grew along the (110) orientations that were perpendicular to the *c* axis in the hybrid materials. The (110)-oriented growth might lead to the formation of thin slabs.^40^ As shown in Fig. S1,† the BiOCl prepared in the presence of different H_3_BTC contents showed different intensities of (110) diffraction peak. A high content of H_3_BTC can improve the (110)-oriented growth. In addition, the iron ions strongly induced BiOCl to preferentially grow along the (110) orientation. These results also suggest that a strong interaction existed between MIL-100(Fe) and BiOCl. In a previous study,^40^ BiOCl nanosheets with thin slabs showed high photocatalytic activity; hence, the hybrid materials prepared *via* the one-step coprecipitation method in the current study may show enhanced catalytic performance.

Fig. 1b shows the FTIR spectra of BiOCl, BiOCl/MIL-100(Fe) hybrids, and MIL-100(Fe). The wide and strong peak at ∼3400 cm^−1^ is ascribed to the stretching vibrations of –OH from absorbed water molecules.^34^ The MIL-100(Fe) spectrum shows adsorption peaks at 1624, 1442, 1371, 760, and 712 cm^−1^. The peak at 1624 cm^−1^ is attributed to the (C–O) bond of carboxylic groups, and the sharp peaks at 1442 and 1371 cm^−1^ are assigned to the asymmetric and symmetric vibration bands of the O–C–O group, respectively. The bands at 760 and 707 cm^−1^ represent the C–H bending vibration of benzene ring.^41^ The peaks at 630 cm^−1^ correspond to the formation of Fe–oxo bond between the carboxylic group and Fe(iii) ions.^42^ The characteristic peak at 510 cm^−1^ is attributed to the stretching vibration of the Bi–O band.^1^ As shown in Fig. 1b, the above-mentioned characteristic peaks existed in BiOCl/MIL-100(Fe) hybrids. However, the peak at 510 cm^−1^, attributed to Bi–O band, in the spectrum of the BiOCl/MIL-100(Fe) hybrid was blueshifted compared with that in the spectrum of BiOCl, which indicates that BiOCl and MIL-100(Fe) were coupled.^1,43^

**Fig. 2:**
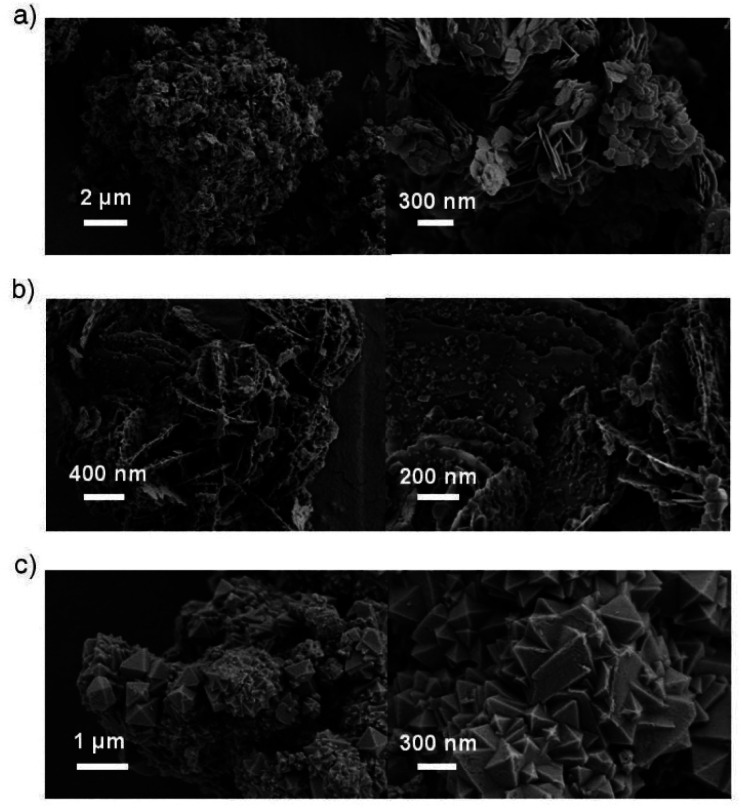
Scanning electron microscopy images of (a) BiOCl, (b) BMF-50, (c) MIL-100(Fe).

The morphologies of the hybrids were investigated *via* SEM, as shown in Fig. 2. The MOF MIL-100(Fe) exhibited larger octahedral crystals together with small particles (Fig. 2c), which agrees with the findings in the literature.^44,45^ However, the MIL-100(Fe) surface featured some roughness, which was likely from the incorporation of CUSs in the MOF.^13^ The pure BiOCl exhibited square sheet-like shape with 20–40 nm thickness. In the BiOCl/MIL-100(Fe) composite, MIL-100(Fe) crystals grew on the BiOCl surface, with BiOCl serving as a support (Fig. 2b), which is consistent with TEM image (Fig. S2†). Particularly, the BiOCl in BMF-50 exhibited flowerlike hierarchical microspheres structure assembled by BiOCl nanosheets, and the MOFs grew on the petaloid BiOCl nanosheet. With the increasing BiOCl content, the size and quantities of MOFs crystal reduced. The detailed morphology of BMF-50 hybrid materials was further obtained *via* TEM (Fig. S2†). The MOF nanoparticles were uniformly distributed in the BiOCl grid. Moreover, BiOCl were composed of nanosheets, and the calculated lattice spacings of 0.738, 0.344, 0.267, and 0.275 nm are assigned to [001], [101], [102], and [110] facets, respectively.^34,46–48^ The SEM and TEM results also prove the successful building of BiOCl/MIL-100(Fe) heterojunctions. The flowerlike hierarchical microspheres assembled by thinner nanosheets have the advantage of higher light-harvesting ability and largely expedite the separation of photoinduced carriers, resulting in enhanced photocatalytic activity of BiOBr heterojunctions; moreover, the exposed (110) active crystal facets will play an important role in achieving excellent photocatalytic activity.^49^ Hence, the BiOCl/MIL-100(Fe) hybrid materials prepared *via* this simple method may exhibit excellent catalytic performance under visible light.

The N_2_ adsorption–desorption isothermal curves and pore-diameter distribution of catalysts were measured. The results depicted in Fig. S3† show that the specific areas of the BiOCl/MIL-100(Fe) hybrids were obviously greater than that of the pure BiOCl, and the hybrids had a mesoporous structure. Meanwhile, the surface areas of the hybrids were lower than that of MIL-100(Fe), which may be due to the presence of BiOCl or residual impurities such as traces of trimesic acid or to the inhibition of the formation of crystalline MIL-100(Fe).^35^ The large surface area and proper aperture increased the potential of BiOCl/MIL-100(Fe) to interact with organic contaminants for catalytic degradation. The presence of mesopores favors multilight scattering/reflection, resulting in enhanced harvesting of the exciting light and thus improved photocatalytic activity.^50,51^ In addition, larger mesopores facilitate mass transport, resulting in improved performance.^52–55^

**Fig. 3:**
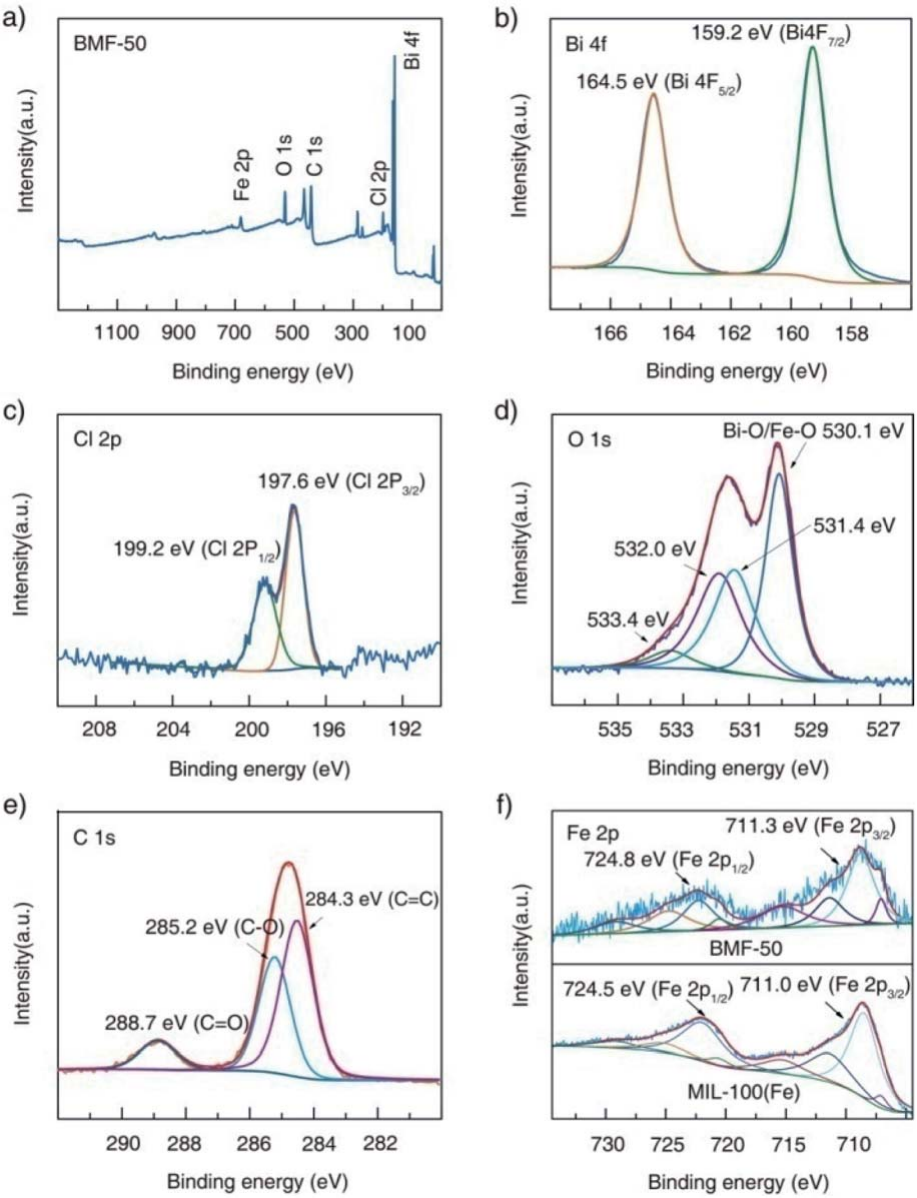
X-ray photoelectron spectra of BMF-50: (a) survey scan, (b) Bi 4f, (c) Cl 2p, (d) O 1s, (e) C 1s; (f) comparison of the Fe 2p spectra of BMF-50 and MIL-100(Fe).

Furthermore, the surface elemental compositions of the as-prepared materials were analyzed *via* XPS. The survey spectrum shown in Fig. 3a indicates the existence of Fe, O, C, Bi, and Cl in the BiOCl/MIL-100(Fe) (BMF-50) hybrids. In the Bi 4f spectrum (Fig. 3b), the peaks at 159.2 eV and 164.2 eV are attributed to Bi 4f_5/2_ and Bi 4f_7/2_, respectively, which are characteristic of the Bi^3+^ in the hybrids.^34^ In the Cl 2p spectrum, two peaks existed at 199.2 and 197.6 eV and are assigned to Cl 2p_1/2_ and Cl 2p_3/2_, respectively. The O1s spectrum of BMF-50 was deconvoluted into four peaks: at 530.1, 531.4, 532.0, and 533.4 eV, which are assigned to the Fe–O and Bi–O bonds, the O atom from terephthalic acid, adsorbed H_2_O or hydroxyl groups on the surface of the hybrids, and oxygen vacancies, respectively.^1,34^ As shown in Fig. 3e, the C 1s spectrum featured three peaks, located at 284.3, 285.2, and 288.7 eV, ascribed to C=C, C–O, and C=O bonds of terephthalic acid, respectively.^34^ The XPS spectra of Fe 2p after Gaussian curve fitting are displayed in Fig. 3f; the spectra featured two typical main peaks of Fe 2p_3/2_ and Fe 2p_1/2_, at 711.3 eV and 724.8 eV, respectively. In addition, the fitted peaks located at 711.3, 713.9, 724.8, and 727.3 eV are ascribed to the Fe^III^ cation, and the two shakeup satellite peaks at about 717.8 and 731.7 eV are the fingerprint of Fe^III^ species, which indicates that the iron in BiOCl/MIL-100(Fe) was predominantly in the Fe^III^ state.^13^ Interestingly, new multiple peaks at 709.6 and 723.1 eV, attributed to Fe^II^ species, appeared in the deconvoluted Fe 2p curves of BMF-50 hybrid materials.^13^ This indicates that some Fe^II^ centers of MIL-100(Fe) were formed during the preparation of the BiOCl/MIL-100(Fe) hybrids, which resulted in the high Fenton catalytic performance of MIL-100(Fe) with Fe^II^ CUS. Moreover, Fe^II^ species were still found in pure MIL-100(Fe), with FeCl_2_ as the ferrous source, while the Fe^II^ species content was lower than those of the BiOCl/MIL-100(Fe) hybrids. This means that the existence of Bi^3+^ affected the formation of MIL-100(Fe). Moreover, two peaks, at 711.0 and 724.5 eV, fitted to Fe 2p_3/2_ and Fe 2p_1/2_ in the Fe 2p spectrum of MIL-100(Fe) showed slight shifts compared with their positions in the spectra of the BiOCl/MIL-100(Fe) hybrids; this also indicates that a strong chemical bonding existed between BiOCl and MIL-100(Fe).^1^ Overall, the above XPS results confirm the successful preparation of BiOCl/MIL-100(Fe) hybrids.

**Fig. 4:**
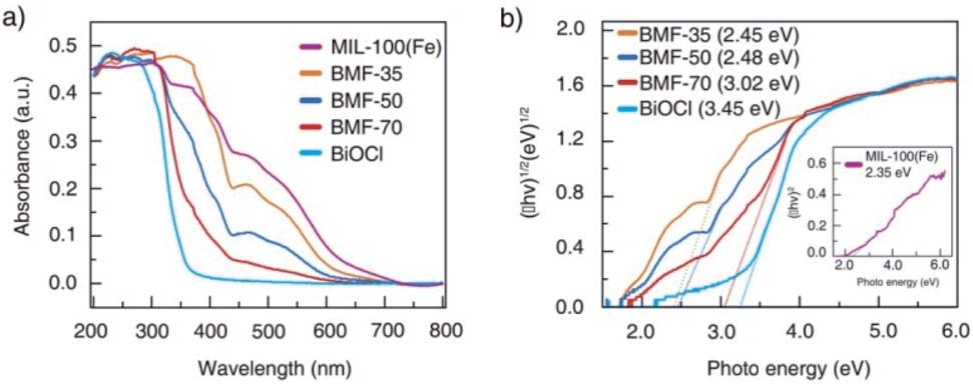
(a) Ultraviolet-visible diffuse reflectance spectra of BiOCl/MIL-100(Fe) hybrid composites; (b) optical bandgap energy of MIL-100(Fe), BiOCl, and hybrids.

The light-response abilities of BiOCl/MIL-100(Fe) hybrids were determined *via* UV-vis DRS (Fig. 4). As depicted in the figure, BiOCl displayed strong light response in the UV region of 200 to 400 nm. In contrast, MIL-100(Fe) exhibited visible light absorption in the range of 200 to 700 nm. The hybrids exhibited a wider absorption edge than BiOCl owing to the strong interaction between BiOCl nanosheet and MIL-100(Fe) through tight chemically bonded interfaces, which resulted in more photogenerated electron–hole pairs and enhanced photocatalytic activity. The band-gap energy (*E*_g_) of these materials can also be estimated *via* Tauc's plots using the following equation:^1^

1 *α*(*hν*) = *A*(*hν* − *E*_g_)^*n*/2^,

where *α*, *h*, *ν*, and *A* are the absorption coefficient, Planck's constant, light frequency, and a constant, respectively. The value of the coefficient *n* is related to the type of optical transition in the semiconductor. The *n* value of BiOCl is 4, which suggests indirect transition, whereas the *n* is 1 for MIL-100(Fe), which indicates that MIL-100(Fe) featured direct optical transition.^1^ The *E*_g_ values of MIL-100(Fe), BMF-35, BMF-50, BMF-70, and BiOCl were estimated as 2.35, 2.45, 2.48, 3.02, and 3.25 eV, respectively (Fig. 4b). The narrowing bandgap of hybrid materials is consistent with the enhanced optical response, and it can be attributed to the formation of chemical bonds between BiOCl and MIL-100(Fe).

### 3.2 Catalytic activity

**Fig. 5:**
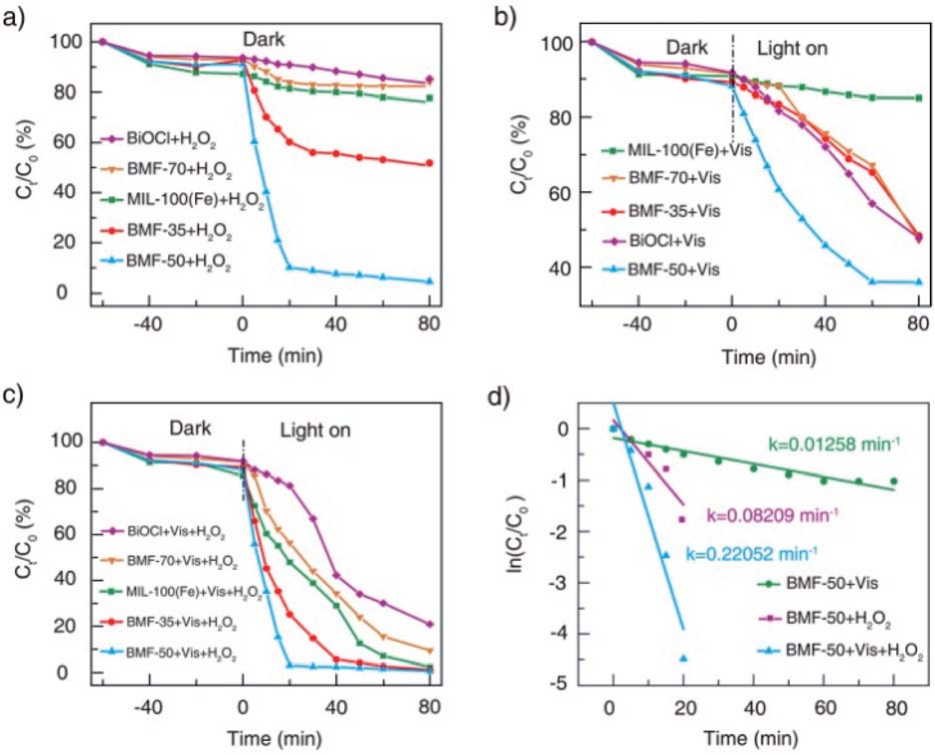
Catalytic degradation of RhB under different conditions (a, b, c); pseudo-first-order kinetics curves of RhB degradation with BMF-50 as catalyst (d).

The catalytic performances of BiOCl/MIL-100(Fe) hybrids were investigated under H_2_O_2_, visible light, and H_2_O_2_ + vis, with RhB as model contaminants. As shown in Fig. 5a, all catalysts showed relatively weak adsorption capability. Without irradiation, the RhB removal rates for the parent materials BiOCl and MIL-100(Fe) were about 10% and 20%, respectively. Meanwhile, the BiOCl/MIL-100(Fe) hybrids (BMF-50) showed the strongest Fenton catalytic performance with H_2_O_2_ as the oxidizing agent: the corresponding RhB removal rate reached 90% within 20 min. The BMF-35 sample also showed increasing Fenton catalytic performance for RhB degradation. As shown in Fig. 5b, the pure MIL-100(Fe) exhibited weak photocatalytic activity without H_2_O_2_ as the oxidizing agent. The RhB removal rate reached 53% within 80 min with pure BiOCl as the catalyst under irradiation. The BiOCl/MIL-100(Fe) hybrids displayed better photocatalytic degradation of RhB than pure MIL-100(Fe); especially, the BMF-50 exhibited the best removal efficiency: the RhB removal rate reached 64% in 60 min. Interestingly, the BMF-50 showed the strongest photo-Fenton catalytic performance: the RhB removal rate reached 97% in 20 min. The BMF-35 also exhibited better photo-Fenton catalytic activity than MIL-100(Fe); the enhanced photo-Fenton catalytic activity can be attributed to the formation of a heterojunction between BiOCl and MIL-100(Fe). An excessive or small number of MOFs in the hybrid materials is not helpful for the separation of photoinduced charges.^56^ To further understand the mechanism behind the improved photo-Fenton catalytic property, the kinetics curves were obtained, and they conformed to the pseudo-first-order kinetics (ln(*C*/*C*_0_) = −*kt*) (Fig. 5d). As shown in Fig. 5d, the dynamic constant corresponding to BMF-50 under vis + H_2_O_2_ was 0.2205 min^−1^, which was 2.7 and 17.5 times those of BMF-50 under H_2_O_2_ and vis, respectively. Furthermore, the degradation of higher concentrations of contaminants in wastewater with hybrid materials as heterogeneous Fenton catalysts was further investigated. Fig. S4† displays the degradation curves of MB for different catalysts when the initial MB concentration was 500 mg L^−1^, catalyst dose was 600 mg L^−1^, H_2_O_2_ amount was 7.44 mmol L^−1^, and initial pH of solution was 4.0. The MB removal rate over BMF-50 reached 99% within 2 min, and the removal efficiency was significantly higher than that over pure MIL-100(Fe) and higher than that over MIL-100(Fe) reported in the literature.^57^ The mineralization of dye was examined based on the TOC removal rate, and the results indicated the extent of oxidative destruction for organic pollutants. The TOC removal rate of MB after 30 min was also investigated. When BMF-50 was used as the catalyst, nearly 62.5% TOC removal was obtained, which was significantly higher than those obtained over MIL-100(Fe) and BiOCl (Fig. S5†). These results further confirm that BiOCl/MIL-100(Fe) showed enhanced catalytic activity in dye degradation.

The reusability and stability of catalysts were also studied by evaluating the recycling ability of BMF-50. As shown in Fig. S6,† the RhB degradation rate was still over 93% after the fifth run, indicating the excellent reusability and stability of BMF-50. Moreover, the XRD patterns and FTIR spectra of BMF-50 after the catalytic reaction were not significantly different from those before the reaction (Fig. S7 and S8†), further proving the catalyst superior reusability and stability. Additionally, the used BMF-50 had higher Fe(ii) content (Fig. S9†), indicating that Fe(ii) was generated on the MIL-100(Fe) surface or BiOCl/MIL-100(Fe) interface, which affected the H_2_O_2_ activation. In summary, the catalytic activity of BMF-50 was higher than those of MIL-100(Fe) and BiOCl respectively, and the BiOCl/MIL-100(Fe) hybrids possess enormous potential to handle environmental problems because of their strong capability of photo-Fenton degradation.

### 3.3 Catalytic degradation mechanism

**Fig. 6:**
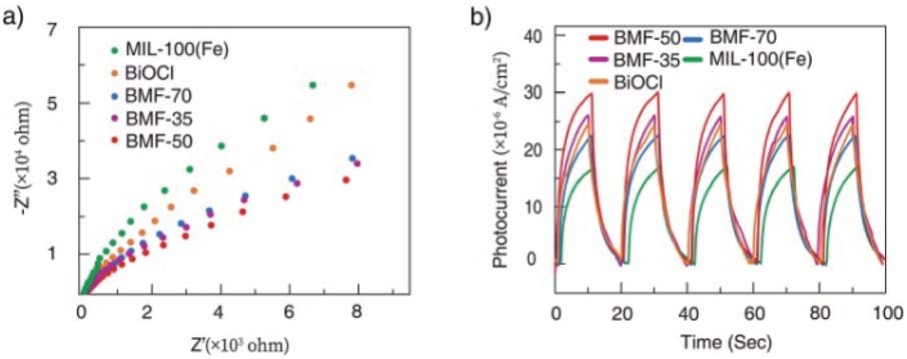
(a) Nyquist impedance plots and (b) transient photocurrent responses of the samples.

To understand the cause of the photo-Fenton catalytic activity of BiOCl/MIL-100(Fe), analyses were conducted using a series of characterization techniques, including Nyquist impedance spectroscopy, transient photocurrent measurements, steady-state PL spectroscopy, and linear sweep voltammetry. Fig. 6a shows the Nyquist plots of different catalysts. The Nyquist semicircle diameter is related to the corresponding electrode impedance. The curve of BMF-50 showed a smaller arc radius than those of the other catalysts, indicating the optimal charge transmission efficiency and electronic conductivity.^1^ Moreover, as depicted by the photocurrent curves (Fig. 6b), the BMF-50 hybrid showed stronger photocurrent response intensity than the other catalysts. This phenomenon suggests that it is beneficial to use BMF-50 as the catalyst for better conduction and separation of the photoinduced electrons and holes and superior visible light utilization. Photoluminescence spectroscopy has been widely applied to analyze the transfer, separation, and recombination of photogenerated carriers. The PL spectra of MIL-100(Fe), BiOCl, and the hybrids under 320 nm excitation wavelength are displayed in Fig. S10.† The hybrid BMF-50 showed the lowest fluorescence emission intensity among all the catalysts, which also indicates that the hybrid has a high efficiency to separate the photoinduced electrons and holes, leading to enhanced photocatalytic performance. In addition, these catalysts were analyzed *via* linear sweep voltammetry. The onset potentials of BiOCl, MIL-100(Fe), BMF-35, BMF-50, and BMF-70 were −0.784, −0.916, −0.662, −0.641, and −0.916 V for achieving the current density of −0.08 mA cm^−2^ (Fig. S11†). Overall, the results reveal that the BiOCl/MIL-100(Fe) hybrid can improve the photocatalyst electrochemical property.^58–60^ According to the characterization results, a heterojunction between BiOCl and MIL-100(Fe) was formed in the hybrid materials, and it is important to transfer and disperse the generated charges to promote the photocatalytic degradation.

**Fig. 7:**
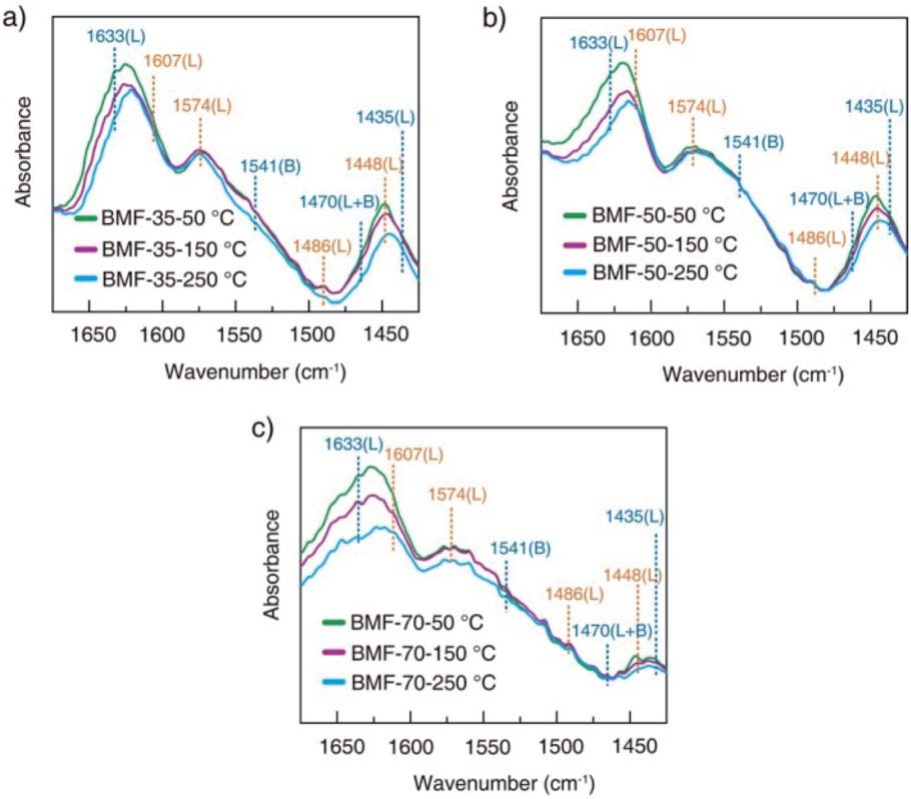
Pyridine-infrared spectral measurements of BiOCl/MIL-100(Fe) at different degassing temperatures (a) 25 °C, (b) 150 °C, and (c) 250 °C.

The rate of Fenton or photo-Fenton reactions in MOFs strongly depends on the exposed active metallic components, which has been confirmed to be the rate-determining step in heterogeneous and homogenous Fenton-like reactions.^61^ Metal–organic frameworks can characteristically act as Lewis acid catalysts owing to the presence of unsaturated or open metal centers. The CUCs are considered the key factor for efficient photo-Fenton activity. Moreover, CUCs in MOFs are the main active sites for Lewis acid-based catalytic reactions.^29^ In addition, H_2_O_2_ behaves as a Lewis base; it tends to be adsorbed on CUCs (Lewis acid sites) with strong affinity, leading to abundant ˙OH production. According to the above results, the BiOCl/MIL-100(Fe) hybrids, especially BMF-50, exhibited enhanced Fenton and photo-Fenton performance, attributed to the fast generation of ˙OH radicals following H_2_O_2_ decomposition. The XPS analysis revealed the occurrence of mixed-valence ferrous iron ions Fe^II^ and Fe^III^ in the MOFs and hybrids, which also suggests that there were a larger number of coordinatively unsaturated iron centers with mixed-valence ions Fe^II^ and Fe^III^. These CUCs behave as Lewis acid sites, facilitating the adsorption and fast decomposition of H_2_O_2_. The high Fenton and photo-Fenton activities of catalysts are generally related to the high acidity of active sites.^29,62–64^ Pyridine as non-reactive vapor is often used as a probe molecule to identify the Lewis acid sites. The medium-strength and strong acid sites can be titrated using this probe molecule. The strength of acid sites can be identified by studying the relationship between probe molecules and acid sites *via* infrared (IR) spectroscopy. In principle, the adsorptions of pyridine onto the CUCs in MOFs will result in the creation of extra IR spectral peaks. The type and strength of surface acid sites on BiOCl/MIL-100(Fe) were evaluated *via* Py-FTIR analysis. Fig. 7 shows the Py-FTIR spectra of hybrid materials under different annealing temperatures. After the physisorbed pyridine was removed under vacuum at different temperatures, the resultant IR spectrum for the catalysts showed three peaks: at 1435 cm^−1^, 1470 cm^−1^, and 1541 cm^−1^, suggesting the occurrences of Lewis, Brønsted + Lewis, and Brønsted acid sites, respectively, from MIL-100(Fe), in the hybrid materials. The peaks at 1435, 1470, and 1633 cm^−1^ can be respectively ascribed to the ν19b, ν19b, and ν8a modes of pyridine coordination to Fe^II^/Fe^III^ CUCs in the MIL-100(Fe).^29,65^ Meanwhile, peaks representing Lewis acid sites on BiOCl (1448, 1486, 1574, and 1607 cm^−1^) were also found.^64,66^ As shown in Fig. 7, the strength of the chemisorption peak of pyridine on the catalyst acid site decreased with the increase in temperature. By calculating the contents and proportions of the weak acid, medium-strength acid, and strong acid under different temperature conditions, the relative contents of Brønsted acid and Lewis acid were analyzed, and the relationship between the catalytic activity and acid sites was analyzed. The calculation formulas of the Brønsted acid and Lewis acid contents are as follows:^67^

2 *C*(pyridine on B sites) = 1.88IA(B)*R*^2^/*W*,

3 *C*(pyridine on L sites) = 1.42IA(L)*R*^2^/*W*,

where *C* is the acid content (mmol g^−1^); IA (B.L) is the comprehensive absorbance (cm^−1^) of the corresponding bands of Brønsted acid or Lewis acid; *R* is the tablet radius (0.65 cm); and *W* is the tablet weight (mg).4
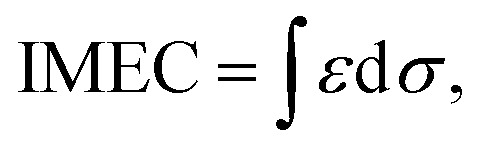

5 *A* = *εCD*,

where *σ* is the wavelength (cm^−1^); *ε* is the molar extinction coefficient (dm^3^ (mol^−1^ cm^−1^)); and *A* is absorbance, defined as log base_10_(*I*_0_/*I*), where *I*_0_ and *I* are the intensity of incident radiation and transmitted radiation; *C* is the concentration (mol dm^−3^); *D* is the path length (cm).

Hence, the number of acid sites on the hybrid materials was quantitatively evaluated (Table S1†). Furthermore, the total acid sites, medium-strength acid sites, and strong-acid sites were determined at pyridine sorption degassing temperatures of 50 °C, 150 °C, and 250 °C. As presented in Table 1, the amounts of total acids and medium-strength acids in BiOCl/MIL-100(Fe) gradually decreased, related to the doping ratio of MIL-100(Fe). The CUC content decreased with the decrease in the MIL-100(Fe) amount. Meanwhile, the concentration of strong acids for BMF-50 (55.16%) was significantly higher than those of BMF-35 (41.00%) and BMF-70 (39.33%), and the total number of strong-acid sites in BMF-50 (0.03517) was also higher than those of the other two catalysts. The results suggest that the hybrid strongly affected the coordination environment of metal ions, further resulting in the change of the type and strength of acid centers on the catalyst surface. Moreover, the higher the strong acid proportion and content, the stronger the catalytic activity of the hybrid materials, which indicates that the catalytic activity of BiOCl/MIL-100(Fe) was determined by the CUC content.

**Table 1:** Number of surface acid sites on different BiOCl/MIL-100(Fe) catalysts, as determined *via* Py-FTIR spectroscopy

Sample	B + L (CUCs) (mmol g^−1^)			
	Total	Medium-strength	Strong	Strong (%)
BMF-35	0.08308	0.03012	0.03407	41.009
BMF-50	0.06375	0.01208	0.03517	55.169
BMF-70	0.03468	0.00661	0.01364	39.331

**Fig. 8:**
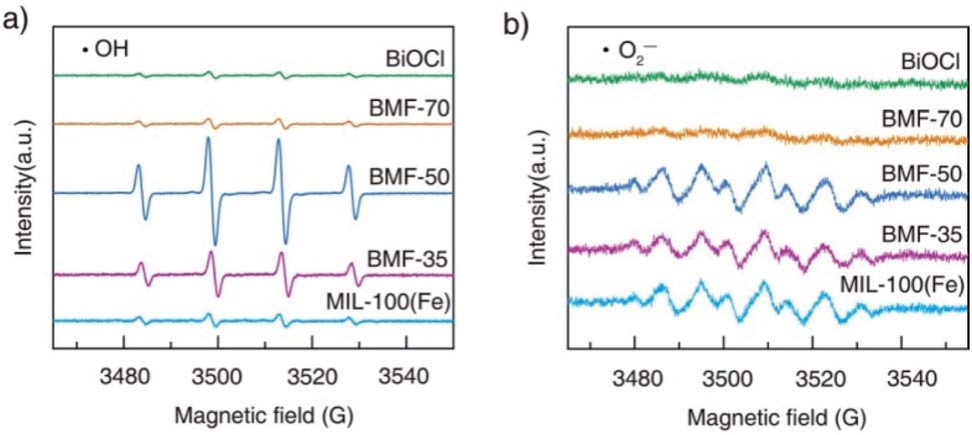
Electron spin resonance spectra of (a) DMPO-˙OH adducts and (b) DMPO-˙O_2_^−^ after 5 min irradiation in the presence of different catalysts.

The reactive oxygen species produced during photo-Fenton catalytic reactions were measured *via* ESR experiments. The generations of ˙OH and ˙O_2_^−^ radicals are demonstrated by the ESR spectra of DMPO-trapped radicals in Fig. 8. As shown in Fig. 8a, strong ESR peaks in the BiOCl/MIL-100(Fe)(BMF-50) spectrum were stronger than those of the other catalysts, suggesting that a higher number of ˙OH radicals were trapped in BMF-50. The ˙OH radical generation of the catalysts followed the order: BMF-50 > BMF-35 > BMF-70 ≈ MIL-100(Fe) > BiOCl. The DMPO-˙O_2_^−^ signals were also detected in the photo-Fenton catalytic process. The peak intensities of the signals for BMF-50 were slightly stronger than those of BMF-35 and MIL-100(Fe) but much stronger than those of BiOCl and BMF-70. The above results suggest that ˙OH may be the main reactive oxidizing species for enhancing RhB degradation. Furthermore, a controlled experiment was performed through the addition of different scavengers. In a series of experiments, *tert*-butanol (TBA), potassium iodide (KI), silver nitrate, and benzoquinone were separately used to quench hydroxyl radicals (˙OH), holes (h^+^), electrons (e^−^), and superoxide free radicals (˙O_2_^−^). As shown in Fig. S12,† the RhB degradation was greatly restricted after TBA introduction. The RhB conversion was also significantly decreased by KI addition, indicating that h^+^ played an important role. In summary, the major active species should be ˙OH radicals, and the h^+^ also had significant effects.

To better understand the energy band structure of BiOCl/MIL-100(Fe), the Mott–Schottky (M–S) experiment was performed in 0.1 M Na_2_SO_4_ solution with pH = 7 at 1000 Hz. As shown in Fig. S13,† the positive slope of the M–S plot suggests that BiOCl and MIL-100(Fe) were n-type semiconductors.^42,68^ The flat band potentials of BiOCl and MIL-100(Fe) could be obtained by extrapolating the lines to 1/*C*^2^ = 0 and were found as −3.68 eV and −0.39 eV (*vs.* Ag/AgCl at pH = 7), respectively. In general, the flat potential is 0.1 V more positive than the conduction band (CB) position for an n-type semiconductor.^69,70^ Therefore, the CB potentials of BiOCl and MIL-100(Fe) were −3.78 eV and −0.49 eV (*vs.* Ag/AgCl at pH = 7), respectively. According to the conversion relationship between normal hydrogen electrode (NHE) and Ag/AgCl electrode, *E*_NHE_ = *E*_Ag/AgCl_ + 0.197,^69^ the CB positions of BiOCl and MIL-100(Fe) were respectively −0.271 eV and −0.29 eV. The valence band potential (*E*_VB_) can be calculated from the empirical equation *E*_g_ = *E*_VB_ − *E*_CB_, where *E*_g_ is the band-gap energy of the semiconductor, and the CB potential (*E*_CB_) is close to the flat band potential.^71^ Correspondingly, the valence band potentials (*E*_VB_) of BiOCl and MIL-100(Fe) were 2.98 eV and 2.06 eV, respectively.

**Fig. 9:**
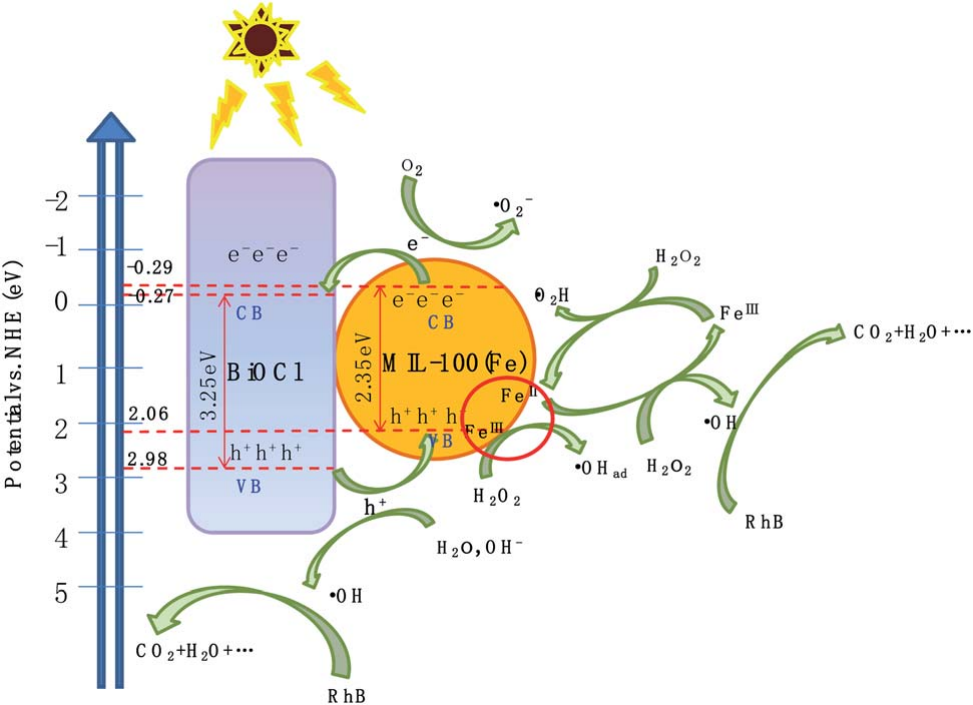
Photo-Fenton catalytic mechanism of BiOCl/MIL-100(Fe) under visible-light illumination.

Based on the above experimental results, a plausible photo-Fenton catalytic degradation mechanism of BiOCl/MIL-100(Fe) is illustrated in Fig. 9. As semiconductor materials, MIL-100(Fe) and BiOCl can generate electron–hole pairs after being excited by electrons. Also, MIL-100(Fe) has a narrower band gap (2.350 eV) than BiOCl (3.240 eV) and can relatively easily absorb visible light to produce electron–hole pairs. Moreover, the CB potential of MIL-100(Fe) (−0.29 eV) was more negative than that of BiOCl (−0.27 eV), and the valence band potential of BiOCl (2.98 eV) was larger than that of MIL-100(Fe) (2.06 eV). Therefore, the electrons in the CB of MIL-100(Fe) were transferred to BiOCl through the heterojunction between BiOCl and MIL-100(Fe). Moreover, the formed intimated interface of the two materials was effective to reduce the recombination of electrons and holes.^72–75^ This unique layered flowerlike structure facilitated the transport of photonic electrons, allowing them to be quickly transferred to the BiOCl. During the photo-Fenton catalytic process, the RhB was adsorbed on the surface of flowerlike BiOCl/MIL-100(Fe) hybrids. After irradiation under simulated sunlight, MIL-100(Fe) and BiOCl were excited to generate e^−^ and h^+^, respectively. Subsequently, the photogenerated electrons (e^−^) on the CB of MIL-100(Fe) with abundant CUCs reduced O_2_ into ˙O_2_^−^. Meanwhile, the light-excited holes on the valence band of BiOCl oxidized H_2_O/OH^−^ to form ˙OH. Ultimately, the ˙OH, O_2_^−^, and holes (h^+^) left on the valence band of MIL-100(Fe) and BiOCl degraded the RhB molecules.

The proposed mechanism for the activation of H_2_O_2_ for RhB degradation over BiOCl/MIL-100(Fe) is illustrated in Fig. 9. Owing to the presence of abundant coordinatively unsaturated iron centers with strong Lewis acids, the pollutants were first adsorbed on the catalyst *via* π–π interaction between MIL-100(Fe) and aromatic rings of RhB, resulting in the enhancement of RhB in the vicinity of coordinatively unsaturated Fe^II^/Fe^III^ centers. After the introduction of H_2_O_2_, the Fe^II^ CUCs reacted with H_2_O_2_ molecules to form Fe^III^ CUCs because of the strong affinity from the strong Lewis acid sites of iron metal with H_2_O_2_ (strong Lewis base). Then, surface-adsorbed ˙OH radicals were produced by the electron transfer between the H_2_O_2_ and Fe^III^. Moreover, Fe^III^ CUCs also could be reduced to Fe^II^ CUCs. The continuous formation of Fe^II^ CUCs and Fe^III^ CUCs occurred during the photo-Fenton reaction, and abundant ˙OH species were generated. This generated ˙OH can eventually degrade pollutants. Moreover, the photogenerated electrons (e) may reduce the Fe^III^ CUCs to Fe^II^ CUCs and thus promote the redox cycle of Fe^3+^/Fe^2+^, resulting in enhanced degradation efficiency. The possible degradation mechanism is outlined by the following equations:

6 BMF-50 + *hν* → BMF-50 (h^+^ + e^−^),

7 h^+^ + H_2_O/OH^−^ → ˙OH,

8 e^−^ + O_2_ → ˙O_2_^−^,

9 Fe^II^ CUCs + H_2_O_2_ → Fe^III^ CUCs + ˙OH + OH^−^,

10 Fe^III^ CUCs + H_2_O_2_ → Fe^II^ CUCs + HO_2_˙ + H^+^,

11 Fe^III^ CUCs + e^−^ → Fe^II^ CUCs,

12 RhB + (˙OH, h^+^ and O_2_^−^) → CO_2_ + H_2_O, …

## 4 Conclusions

In summary, BiOCl/MIL-100(Fe) hybrid materials were prepared *via* a one-pot coprecipitation method. The hybrid materials were used as photo-Fenton catalysts to remove organic pollutants in wastewater. Compared with MIL-100(Fe) and BiOCl, the hybrids, especially BMF-50, showed remarkably enhanced photo-Fenton activity in RhB degradation. This can be ascribed to the presence of coordinatively unsaturated iron centers (Fe^2+^/Fe^3+^-CUCs), abundant Lewis acid sites, and efficient carrier separation on thin BiOCl nanosheets due to the high charge carrier mobility of the nanosheets. The Fe^2+^/Fe^3+^-CUCs in MIL-100(Fe) could substantially improve the catalytic photo-Fenton performance and H_2_O_2_ activation rate for pollutant degradation. The flowerlike BiOCl nanosheets as a co-catalyst could significantly inhibit the recombination of the photoinduced electron–hole pairs. Furthermore, the photo-Fenton mechanism was further studied, and the results showed that ˙OH and h^+^ were the main active species. The coprecipitation-based hybridization approach presented in this paper opens up an avenue for the sustainable fabrication of photo-Fenton catalysts with abundant CUCs and efficient electron–hole separation capacity.

## Conflicts of interest

There are no conflicts to declare.

## Supplementary Material

RA-011-D1RA06549A-s001

## References

[cit1] Tang L., Lv Z., Xue Y., Xu L., Qiu W., Zheng C. (2019). Chem. Eng. J..

[cit2] Mak C. H., Han X., Du M., Kai J.-J., Tsang K. F., Jia G., Cheng K.-C., Shenk H.-H., Hsu H.-Y. (2021). J. Mater. Chem. A.

[cit3] Jing J., Cao C., Ma S., Li Z., Qu G., Xie B., Jin W., Zhao Y. (2021). Chem. Eng. J..

[cit4] Oh W.-D., Lim T.-T. (2019). Chem. Eng. J..

[cit5] Cheng M., Lai C., Liu Y., Zeng G., Huang D., Zhang C., Qin L., Hu L., Zhou C., Xiong W. (2018). Coord. Chem. Rev..

[cit6] Zhao R., Tian Y., Li S., Ma T., Lei H., Zhu G. (2019). J. Mater. Chem. A.

[cit7] Li Y., Qi X., Li G., Wang H. (2021). Chem. Eng. J..

[cit8] Shen S., Lin Z., Song K., Wang Z., Huang L., Yan L., Meng F., Zhang Q., Gu L., Zhong W. (2021). Angew. Chem., Int. Ed..

[cit9] Wang X., Zhang X., Zhang Y., Wang Y., Sun S.-P., Wu W. D., Wu Z. (2020). J. Mater. Chem. A.

[cit10] Zhu H., Liu D., Zou D., Zhang J. (2018). J. Mater. Chem. A.

[cit11] Ai L., Zhang C., Li L., Jiang J. (2014). Appl. Catal. B Environ..

[cit12] Chi H., Wan J., Ma Y., Wang Y., Ding S., Li X. (2019). J. Hazard. Mater..

[cit13] Tang J., Wang J. (2018). Environ. Sci. Technol..

[cit14] Kirchon A., Zhang P., Li J., Joseph E., Chen W., Zhou H. (2020). ACS Appl. Mater. Interfaces.

[cit15] Zhong Z., Li M., Fu J., Wang Y., Muhamm Y., Li S., Wang J., Zhao Z., Zhao Z. (2020). Chem. Eng. J..

[cit16] Lee J., Farha O. K., Roberts J., Scheidt K. A., Nguyen S. T., Hupp J. T. (2009). Chem. Soc. Rev..

[cit17] Yang X., Zhang S., Li P., Gao S., Cao R. (2020). J. Mater. Chem. A.

[cit18] Wu Q., Yang H., Kang L., Gao Z., Ren F. (2020). Appl. Catal. B Environ..

[cit19] Wang Y., Zhong Z., Muhammad Y., He H., Zhao Z., Nie S., Zhao Z. (2020). Chem. Eng. J..

[cit20] Wang Y., Zhong Z., Muhammad Y., He H., Zhao Z., Nie S., Zhao Z. (2020). Chem. Eng. J..

[cit21] Mei W., Song H., Tian Z., Zuo S., Li D., Xu H., Xia D. (2019). Mater. Res. Bull..

[cit22] Nguyen H.-T. T., Dinh V.-P., Phan Q.-A. N., Tran V. A., Doan V.-D., Lee T., Nguyen T. D. (2020). Mater. Lett..

[cit23] Wu Q., Siddique M. S., Yu W. (2021). J. Hazard. Mater..

[cit24] Abdpour S., Kowsaria E., Moghaddam M. R. A. (2018). J. Solid State Chem..

[cit25] Huang W., Liu N., Zhang X., Wu M., Tang L. (2017). Appl. Surf. Sci..

[cit26] Liu N., Huang W., Zhang X., Tang L., Wang L., Wang Y., Wu M. (2018). Appl. Catal. B Environ..

[cit27] Liu N., Huang W., Tang M., Yin C., Gao B., Li Z., Tang L., Lei J., Cui L., Zhang X. (2019). Chem. Eng. J..

[cit28] Zhang Q., Liu J.-B., Chen L., Xiao C.-X., Chen P., Shen S., Guo J.-K., Au C.-T., Yin S.-F. (2020). Appl. Catal. B Environ..

[cit29] Ahmad M., Quan X., Chen S., Yu H. (2020). Appl. Catal. B Environ..

[cit30] Wu J., Xie Y., Ling Y., Si J., Li X., Wang J., Ye H., Zhao J., Li S., Zhao Q., Hou Y. (2020). Chem. Eng. J..

[cit31] Jiao W., Xie Y., He F., Wang K., Ling Y., Hu Y., Wang J., Ye H., Wu J., Hou Y. (2021). Chem. Eng. J..

[cit32] Zhu S.-R., Liu P.-F., Wu M.-K., Zhao W.-N., Li G.-C., Tao K., Yi F.-Y., Han L. (2016). Dalton Trans..

[cit33] Xue Y., Wang P., Wang C., Ao Y. (2018). Chemosphere.

[cit34] Miao S., Zha Z., Li Y., Geng X., Yang J., Cui S., Yang J. (2019). J. Photochem. Photobiol., A.

[cit35] Chávez A. M., Rey A., López J., Álvarez P. M., Beltrán F. J. (2021). Sep. Purif. Technol..

[cit36] Mohammadifard Z., Saboori R., Mirbagheri N. S., Sabbaghi S. (2019). Environ. Pollut..

[cit37] Guesh K., Caiuby C. A. D., Mayoral Á., Díaz-García M., Díaz I., Sanchez-Sanchez M. (2017). Cryst. Growth Des..

[cit38] Chaturvedi G., Kaur A., Umar A., Khan M. A., Algarni H., Kansal S. K. (2020). J. Solid State Chem..

[cit39] Gong Q., Liu Y., Dang Z. (2019). J. Hazard. Mater..

[cit40] Liu J., Chen L., Zhang S., Zhao H. (2018). Mater. Lett..

[cit41] Nehra M., Dilbaghi N., Singhal N. K., Hassan A. A., Kim K.-H., Kumar S. (2019). Environ. Res..

[cit42] Tian H., Araya T., Li R., Fang Y., Huang Y. (2019). Appl. Catal. B Environ..

[cit43] Tong X., Yang Z., Feng J., Li Y., Zhang H. (2018). Appl. Organomet. Chem..

[cit44] Tang J., Wang J. (2017). RSC Adv..

[cit45] Lv H., Zhao H., Cao T., Qian L., Wang Y., Zhao G. (2015). J. Mol. Catal. Chem..

[cit46] Jia Z., Li T., Zheng Z., Zhang J., Liu J., Li R., Wang Y., Zhang X., Wang Y., Fan C. (2020). Chem. Eng. J..

[cit47] Yang C., Zhong J., Li J., Huang S., Duan R. (2020). Mater. Lett..

[cit48] Xie F., Li R., Zhang X., Wang Y., Fan C. (2020). Mater. Lett..

[cit49] Qin Q., Guo Y., Zhou D., Yang Y., Guo Y. (2016). Appl. Surf. Sci..

[cit50] Fang B., Bonakdarpour A., Reilly K., Xing Y., Taghipour F., Wilkinson D. P. (2014). ACS Appl. Mater. Interfaces.

[cit51] Fang B., Xing Y., Bonakdarpour A., Zhang S., Wilkinson D. P. (2015). ACS Sustainable Chem. Eng..

[cit52] Fang B., Kim M., Kim J. H., Yu J.-S. (2008). Langmuir.

[cit53] Fang B., Fan S.-Q., Kim J. H., Kim M.-S., Kim M., Chaudhari N. K., Ko J., Yu J.-S. (2010). Langmuir.

[cit54] Wang Y.-J., Fang B., Li H., Bi X. T., Wang H. (2016). Prog. Mater. Sci..

[cit55] Xing Y., Fang B., Bonakdarpour A., Zhang S., Wilkinson D. P. (2014). Int. J. Hydrogen Energy.

[cit56] Wang H., Yuan X., Wu Y., Zeng G., Chen X., Leng L., Li H. (2015). Appl. Catal. B Environ..

[cit57] Martínez F., Leo P., Orcajo G., Díaz-García M., Sanchez-Sanchez M., Callej G. (2018). Catal. Today.

[cit58] Li W., He S.-A., Ma Q., Wang X., Zhao C.-H. (2019). Appl. Surf. Sci..

[cit59] Sun C., Xu Q., Xie Y., Ling Y., Hou Y. (2018). J. Mater. Chem. A.

[cit60] Hu D., Xie Y., Liu L., Zhou P., Zhao J., Xu J., Ling Y. (2016). Appl. Catal. B Environ..

[cit61] Dhakshinamoorthy A., Alvaro M., Garcia H. (2012). Chem. Commun..

[cit62] Yu D., Wu M., Hu Q., Wang L., Lv C., Zhang L. (2019). J. Hazard. Mater..

[cit63] Yu D., Wang L., Yang T., Yang G., Wang D., Ni H., Wu M. (2021). Chem. Eng. J..

[cit64] He W., Li Z., Lv S., Niu M., Zhou W., Li J., Lu R., Gao H., Pan C., Zhang S. (2021). Chem. Eng. J..

[cit65] Kumar V. V., Naresh G., Sudhakar M., Anjaneyulu C., Bhargava S. K., Tardio J., Reddy V. K., Padmasri A. H., Venugopal A. (2016). RSC Adv..

[cit66] Xu C., Gu F., Wu H. (2017). Appl. Clay Sci..

[cit67] Emeis C. A. (1993). J. Catal..

[cit68] Zhao C., Pan X., Wang Z., Wang C.-C. (2021). Chem. Eng. J..

[cit69] Shao Z., Zhang D., Li H., Su C., Pu X., Geng Y. (2019). Sep. Purif. Technol..

[cit70] Saha S., Das G., Thote J., Banerjee R. (2014). J. Am. Chem. Soc..

[cit71] Liang R., Jing F., Shen L., Qin N., Wu L. (2015). J. Hazard. Mater..

[cit72] Wang Z., Xiao B., Lin Z., Xu Y., Lin Y., Meng F., Zhang Q., Gu L., Fang B., Guo S., Zhong W. (2021). Angew. Chem., Int. Ed..

[cit73] Wang Z., Lin Z., Deng J., Shen S., Meng F., Zhang J., Zhang Q., Zhong W., Gu L. (2021). Adv. Energy Mater..

[cit74] Lin Z., Xiao B., Wang Z., Tao W., Shen S., Huang L., Zhang J., Meng F., Zhang Q., Gu L., Zhong W. (2021). Adv. Funct. Mater..

[cit75] Hussain M. Z., Yang Z., Linden B., Huang Z., Jia Q., Cerrato E., Fischer R. A., Kapteijn F., Zhu Y., Xia Y. (2021). J. Energy Chem..

