# Supplementary material for: Highly efficient heterogeneous photo-Fenton BiOCl/MIL-100(Fe) nanoscaled hybrid catalysts prepared by green one-step coprecipitation for degradation of organic contaminants
Source: RSC Adv. 2021 Oct 1;11(51):32383–93. doi: 10.1039/d1ra06549a (PMC9041883; doi:10.1039/d1ra06549a)
Supplement: RA-011-D1RA06549A-s001 [file RA-011-D1RA06549A-s001.pdf]

Electronic Supplementary Material (ESI) for RSC Advances.
This journal is © The Royal Society of Chemistry 2021

Supplemental Materials

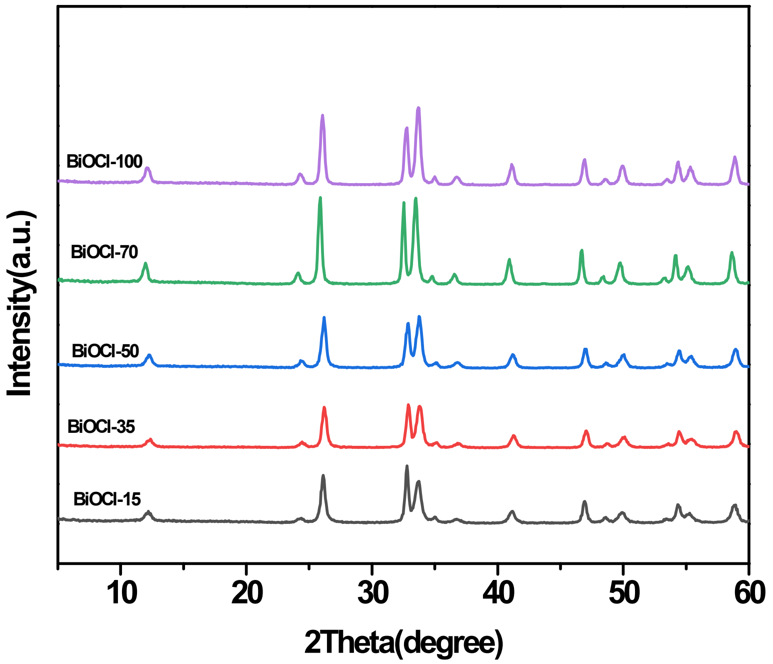

Fig. S1. XRD pattern of BiOCl prepared with existence of H3BTC.

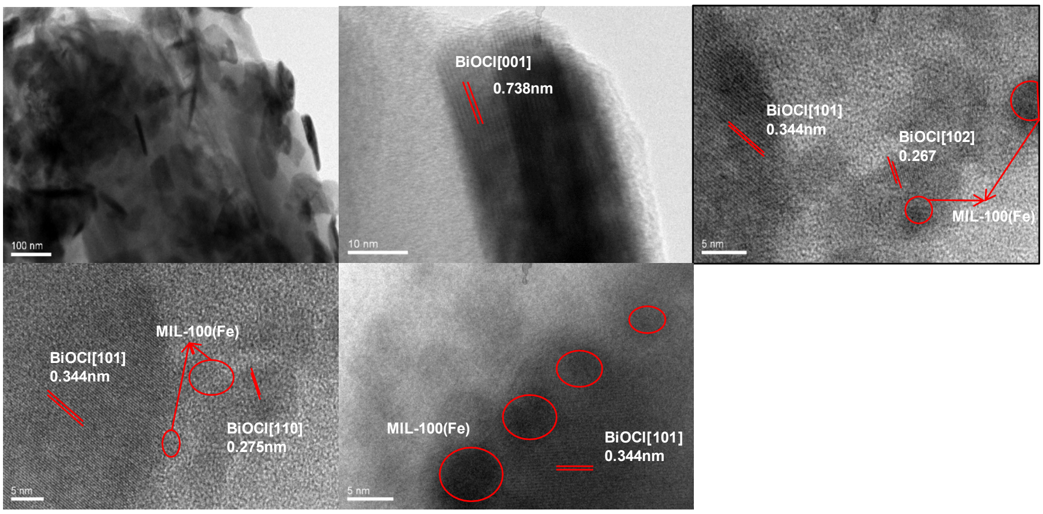

Fig. S2. TEM image of BMF-50.

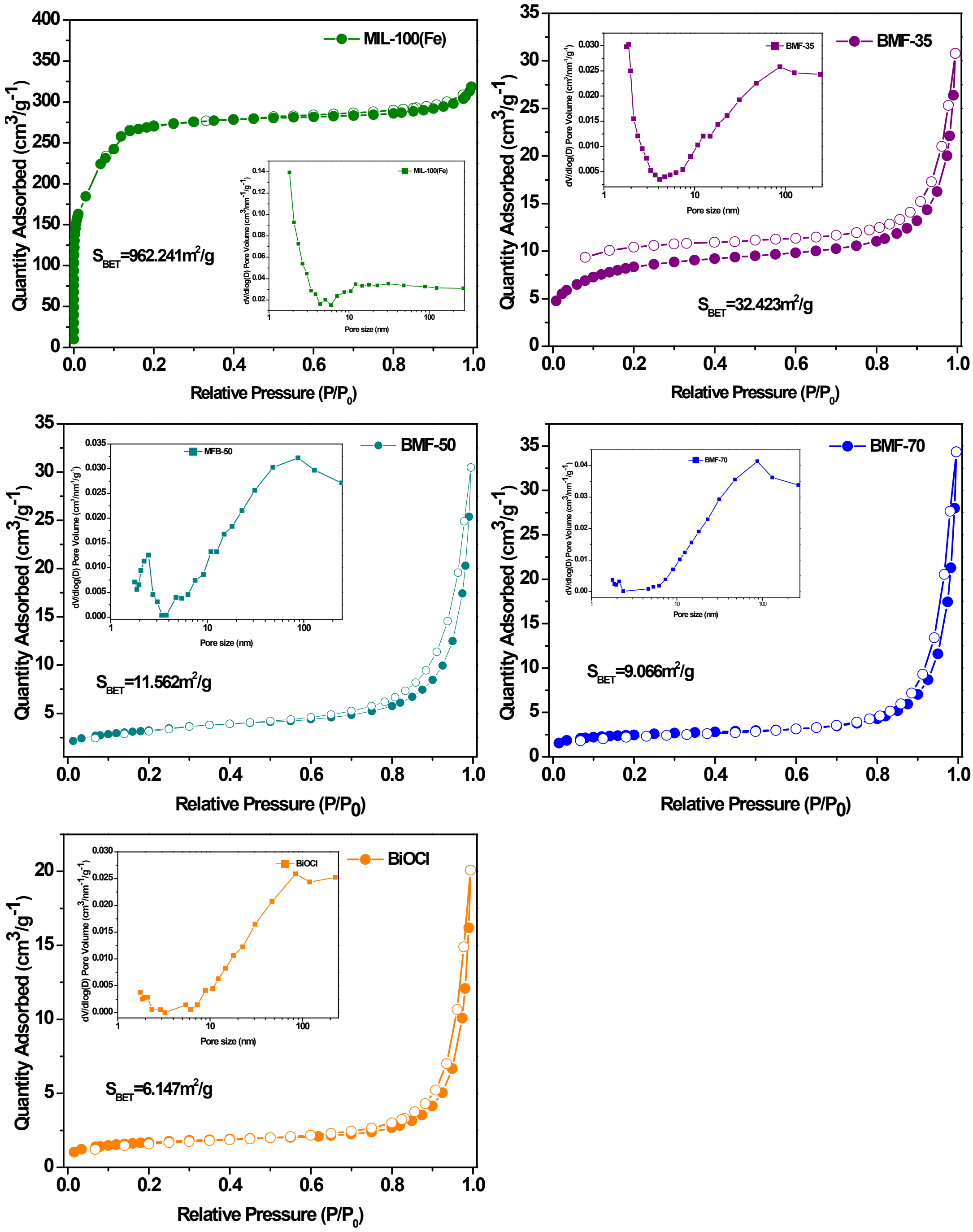

Fig. S3. Nitrogen adsorption-desorption isotherm and the pore size distribution curves for MIL-100(Fe), BMF-35, BMF-50, BMF-70, and BiOCl.

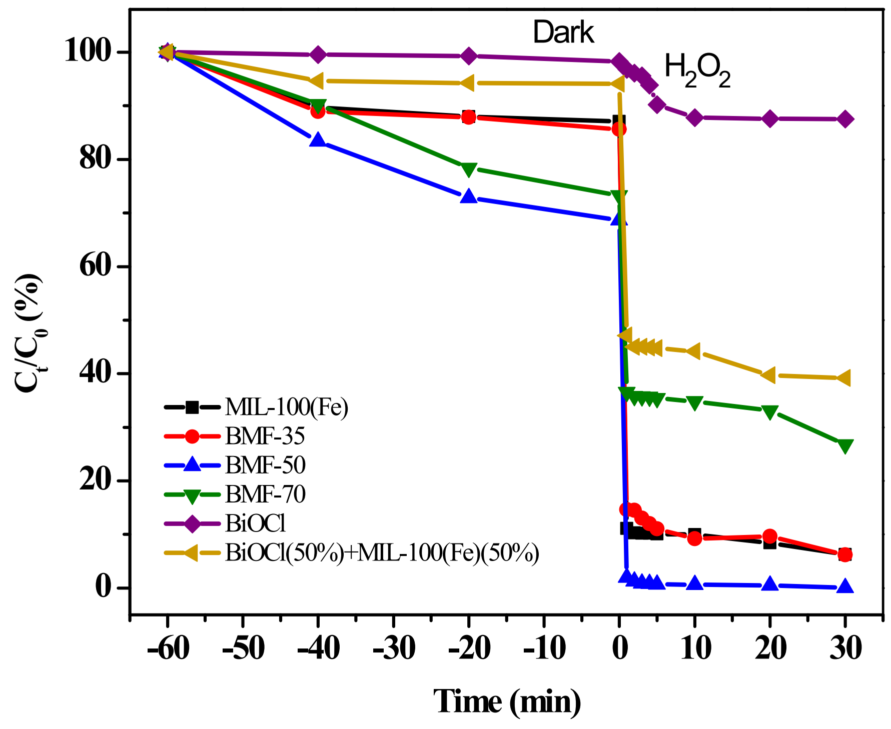

Fig. S4. The adsorption and degradation curves of MB using pure MIL-100(Fe), BiOCl, and BiOCl/MIL-100(Fe) hybrid materials as catalysts. (T = 30°C, $[H_2O_2]_0$ = 7.4 mmol/L, [catalyst] = 600 mg/L, $[Dye]_0$ = 500 mg/L, pH=4.0)

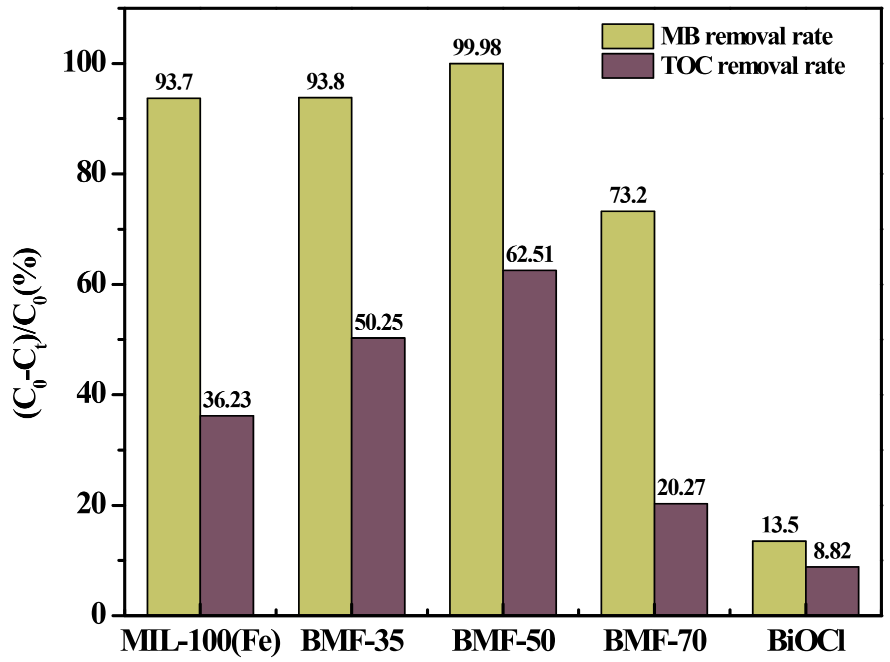

Fig. S5. TOC removal rate of MB with different catalysts after reacting for 30 min.

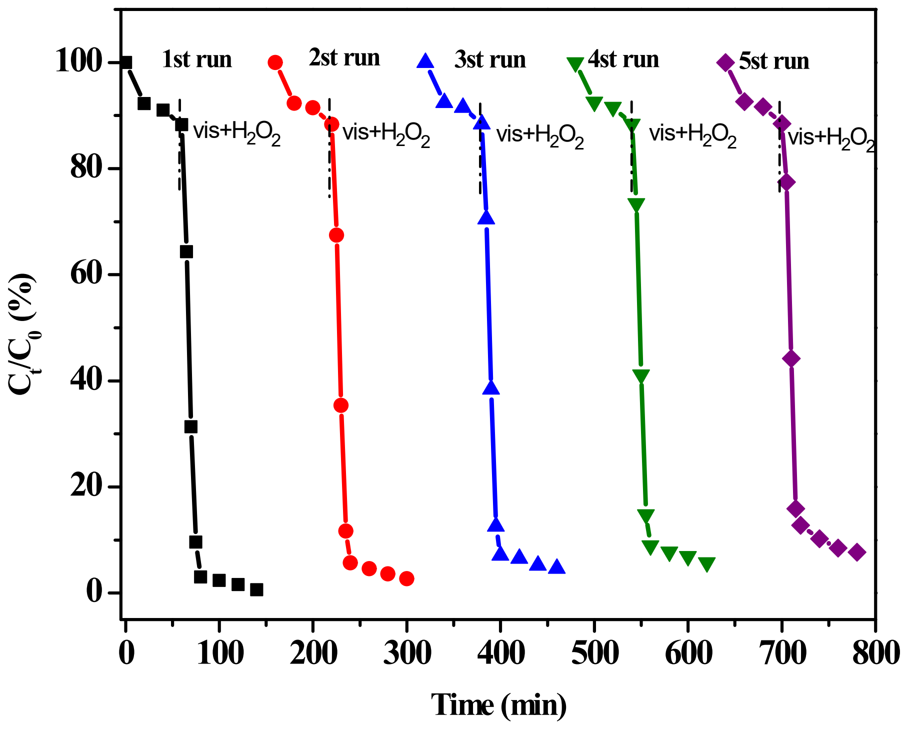

Fig. S6. Reusability of BMF-50 catalyst under the same conditions (T = 30°C $(H_2O_2)_0$ =7.44 mmol/L, (catalyst) = 80 mg/L, $[RhB]_0$ = 40 mg/L)

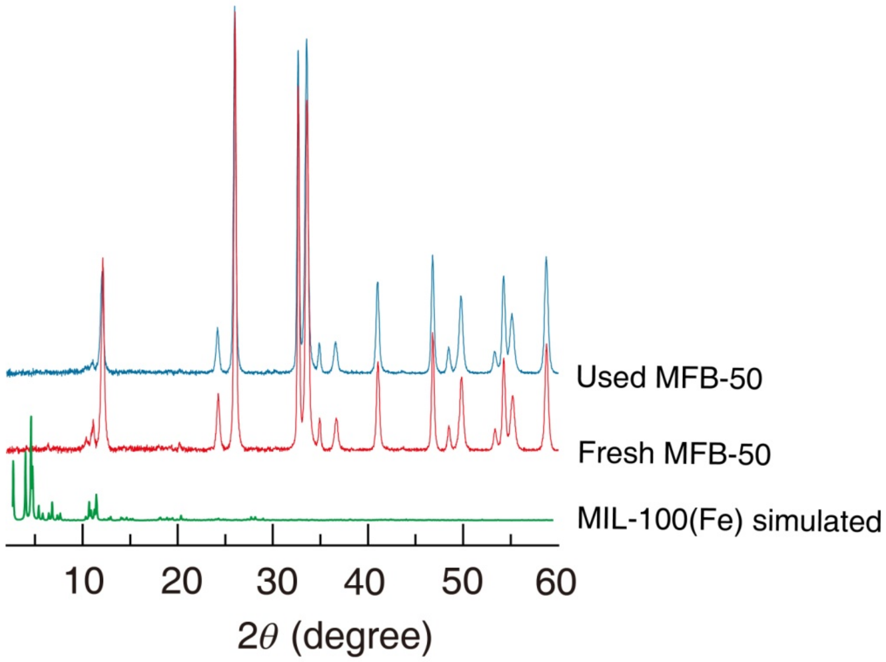

Fig. S7. XRD pattern of BMF-50 (fresh, used).

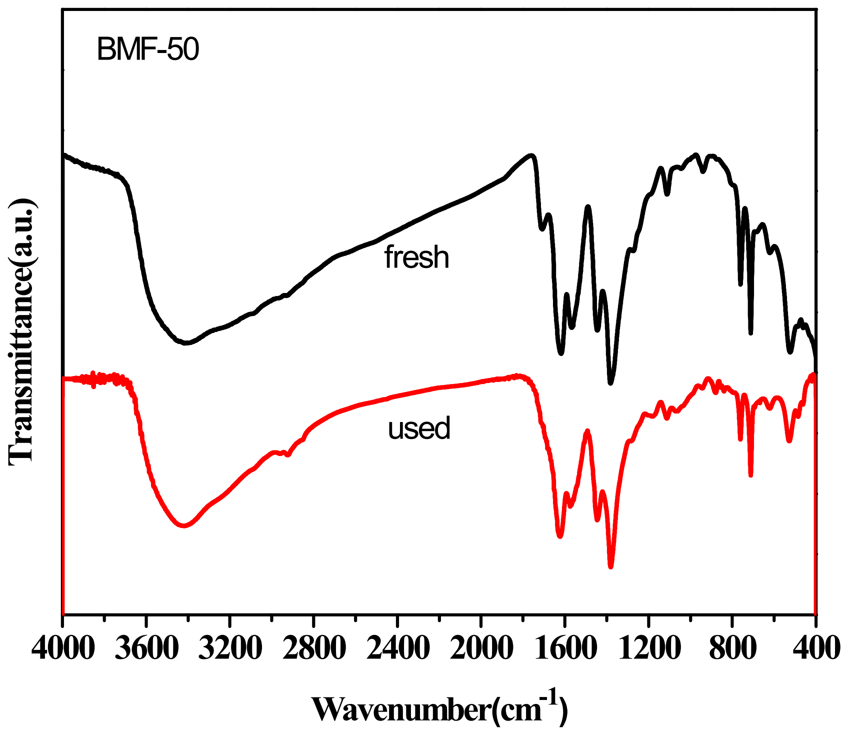

Fig. S8. FT-IR spectra of BMF-50 (fresh, used).

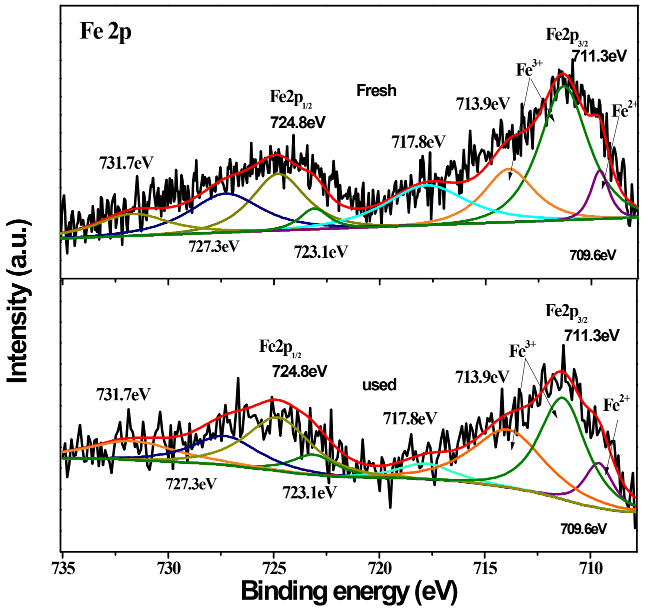

Fig. S9. Fe 2p XPS spectra of BMF-50 (fresh and used).

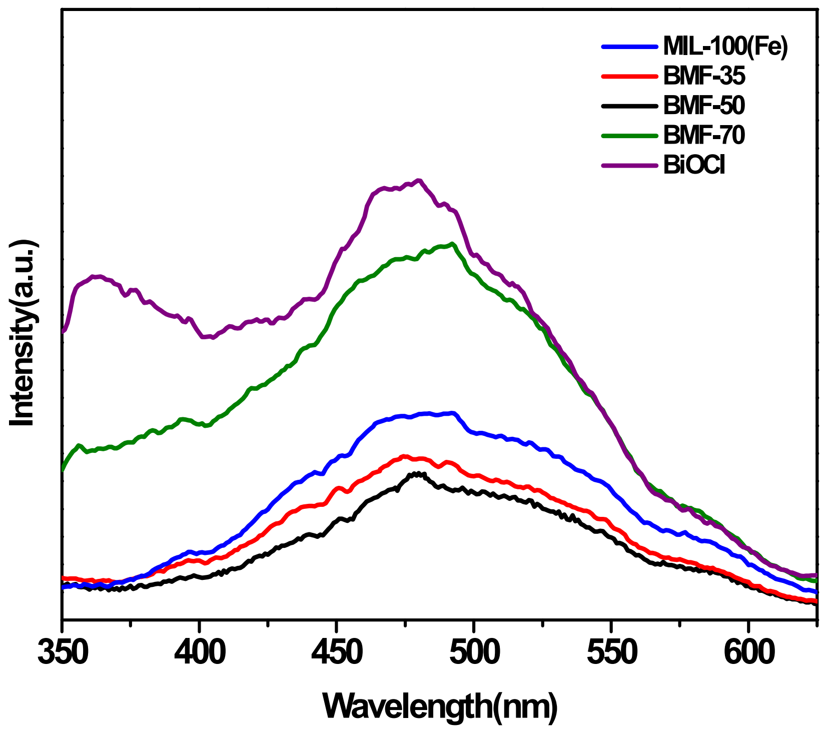

Fig. S10. PL spectra of the different composites.

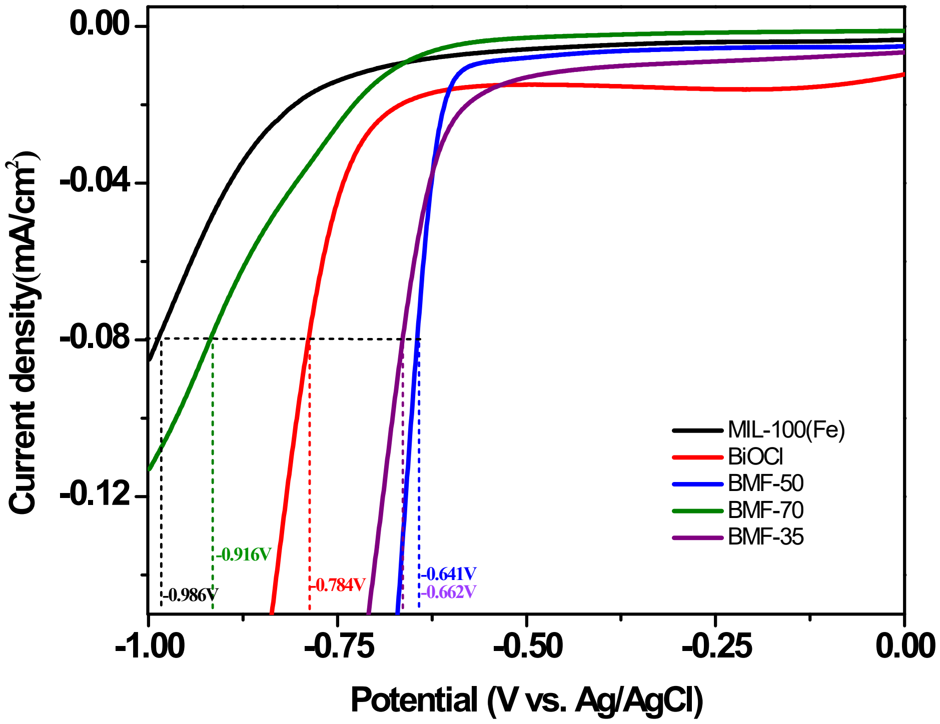

Fig. S11. LSV curves and onset potential of the samples to achieve the current density of −0.08 mA/cm^2.

Table S1. The types and contents of BMF-(35,50,70) surface acids at different temperatures determined by Py -FTIR spectroscopy.

Sample	50°C		150°C		250°C	
	B	L	B	L	B	L
BMF-35	0.01623	0.06685	0.01362	0.05057	0.00579	0.02828
BMF-50	0.01747	0.04628	0.01725	0.03000	0.01203	0.02314
BMF-70	0.01668	0.01800	0.01339	0.00686	0.01021	0.00343

Unit: mmol/g

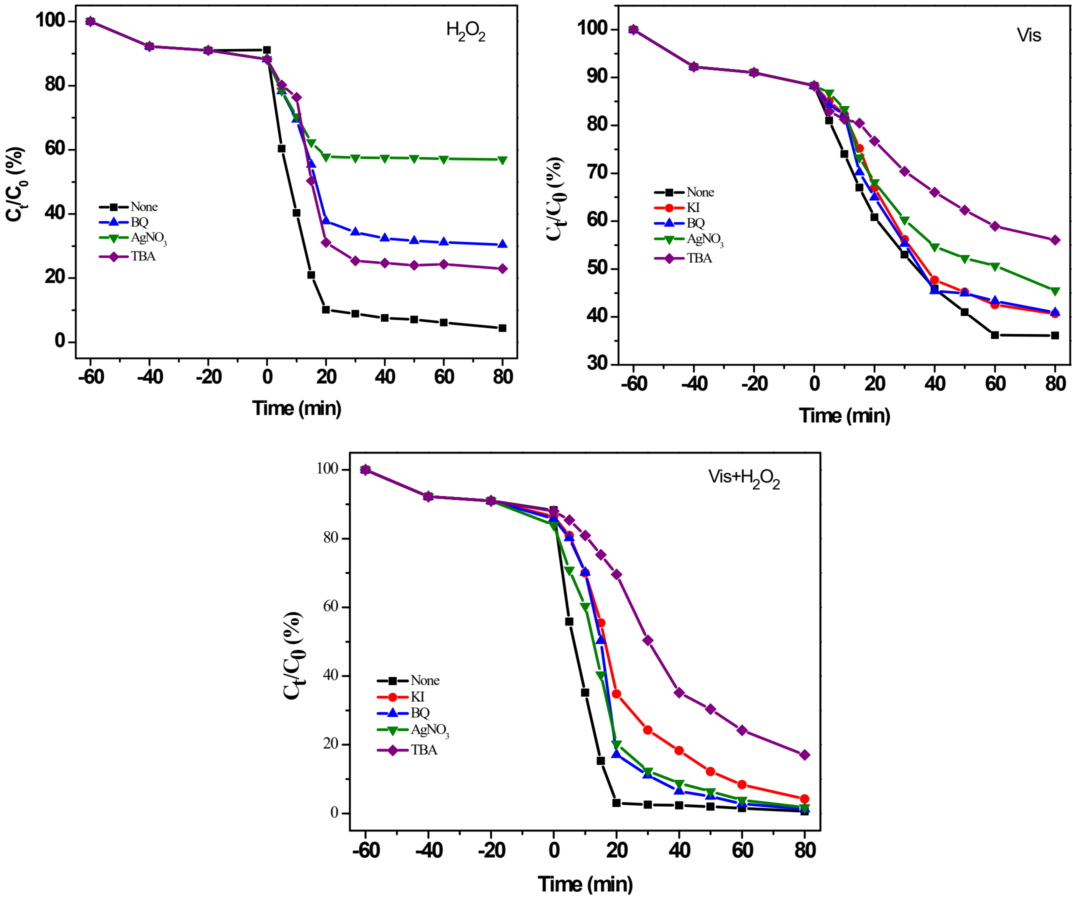

Fig. S12. RhB degradations by BMF-50 under visible light irradiation and H_2O_2 in the presence of several scavenger.

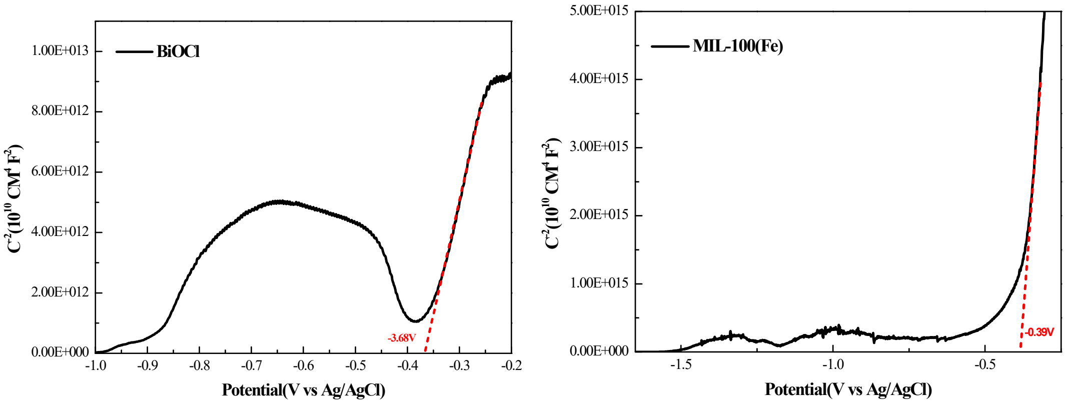

Fig. S13. Motschottky diagrams of MIL-100(Fe) and BiOCl.
